# Naa50/San-dependent N-terminal acetylation of Scc1 is potentially important for sister chromatid cohesion

**DOI:** 10.1038/srep39118

**Published:** 2016-12-20

**Authors:** Ana Luisa Ribeiro, Rui D. Silva, Håvard Foyn, Margarida N. Tiago, Om Singh Rathore, Thomas Arnesen, Rui Gonçalo Martinho

**Affiliations:** 1 Departamento de Ciências Biomédicas e Medicina, Universidade do Algarve, Campus de Gambelas, 8005-139 Faro, Portugal; 2 Center for Biomedical Research (CBMR), University of Algarve, 8005-139 Faro, Portugal; 3 Department of Molecular Biology, University of Bergen, N-5020 Bergen, Norway; 4 Department of Surgery, Haukeland University Hospital, N-5021 Bergen, Norway; 5 Instituto de Medicina Molecular, Faculdade de Medicina, Universidade de Lisboa, Lisboa, Portugal

## Abstract

The gene *separation anxiety (san*) encodes Naa50/San, a N-terminal acetyltransferase required for chromosome segregation during mitosis. Although highly conserved among higher eukaryotes, the mitotic function of this enzyme is still poorly understood. Naa50/San was originally proposed to be required for centromeric sister chromatid cohesion in *Drosophila* and human cells, yet, more recently, it was also suggested to be a negative regulator of microtubule polymerization through internal acetylation of beta Tubulin. We used genetic and biochemical approaches to clarify the function of Naa50/San during development. Our work suggests that Naa50/San is required during tissue proliferation for the correct interaction between the cohesin subunits Scc1 and Smc3. Our results also suggest a working model where Naa50/San N-terminally acetylates the nascent Scc1 polypeptide, and that this co-translational modification is subsequently required for the establishment and/or maintenance of sister chromatid cohesion.

The narrow dimension of the ribosome exit tunnel precludes large domain folding of the nascent protein, which creates a window of opportunity for co-translational modification of protein residues that would be otherwise inaccessible by protein folding^1^. Co-translational modifications occur in archaea, eubacteria, and eukaryotic cells. Among others, they include protein N-terminal acetylation (Nt-Ac)^2^,^3^,^4^,^5^, which involves the transfer of an acetyl group from acetyl-CoA to the protein alpha-amino group^4^,^5^. Nt-Ac is an ubiquitous modification, and partially or fully N-terminally acetylated proteins constitute approximately 50–70% of the proteome in budding yeast, 70–80% in *Drosophila* and 80–90% in human and *Arabidopsis*^6^,^7^,^8^,^9^,^10^. Recent investigations revealed that Nt-Ac might have a major influence on protein stability, complex formation, folding, and subcellular targeting^5^,^11^,^12^,^13^,^14^,^15^,^16^,^17^, as well as being essential for development of multicellular organisms^18^.

Nt-Ac is catalyzed by a highly conserved family of N-terminal acetyltransferases (NATs)^5^. Multicellular eukaryotes have six distinct but highly conserved NATs (NatA to NatF) that most likely were already present in the last eukaryotic common ancestor (LECA)^19^. While some of these NATs are protein complexes requiring different catalytic and auxiliary subunits, other NATs are able to N-terminally acetylate independently of protein partners^10^,^20^,^21^,^22^,^23^,^24^,^25^,^26^,^27^,^28^,^29^,^30^. NATs have distinct substrate specificities and their substrate recognition depends on the nature of the first 2–5 amino acids of the elongating polypeptide^7^,^10^,^31^,^32^,^33^.

The gene *separation anxiety (san*) encodes Naa50/San, the catalytic subunit of NatE, a highly conserved NAT with a classical GNAT fold that acetylates N-termini starting with Methionine followed by hydrophobic (Met-Leu-, Met-Phe-, etc.) or small polar amino acids (Met-Ser-, Met-Thr-, etc.)^21^,^33^,^34^,^35^. It may act physically associated with the NatA subunits Naa10 and Naa15, or independently of this complex^19^,^33^,^35^,^36^,^37^,^38^,^39^. Human and *Drosophila* cells mutant for Naa50/San show chromosome segregation defects during mitosis, including chromosome lagging and chromosomal bridges^36^,^39^,^40^. Interestingly, *Drosophila* Naa50/San is not required for mitosis in the female germ-line stem cells^40^. Naa50/San was originally described as being required in *Drosophila* and human cells for centromeric sister chromatid cohesion^36^,^39^, but more recently, it was also suggested in humans that this enzyme negatively regulates microtubule polymerization through the internal acetylation of beta Tubulin^41^.

In this manuscript we aimed to define the function of Naa50/San during development. Our results suggest that during tissue proliferation Naa50/San behaves as general regulator of sister chromatid cohesion, as it facilitates the correct interaction between cohesin subunits Scc1/Rad21/Vtd and Smc3. Our results also suggest a working model where Naa50/San N-terminally acetylates the nascent Scc1 polypeptide, which is subsequently required for the correct establishment and maintenance of sister chromatid cohesion.

## Results.

### Naa50/San is required for sister chromatid cohesion during *Drosophila* wing development

**Figure 1:**
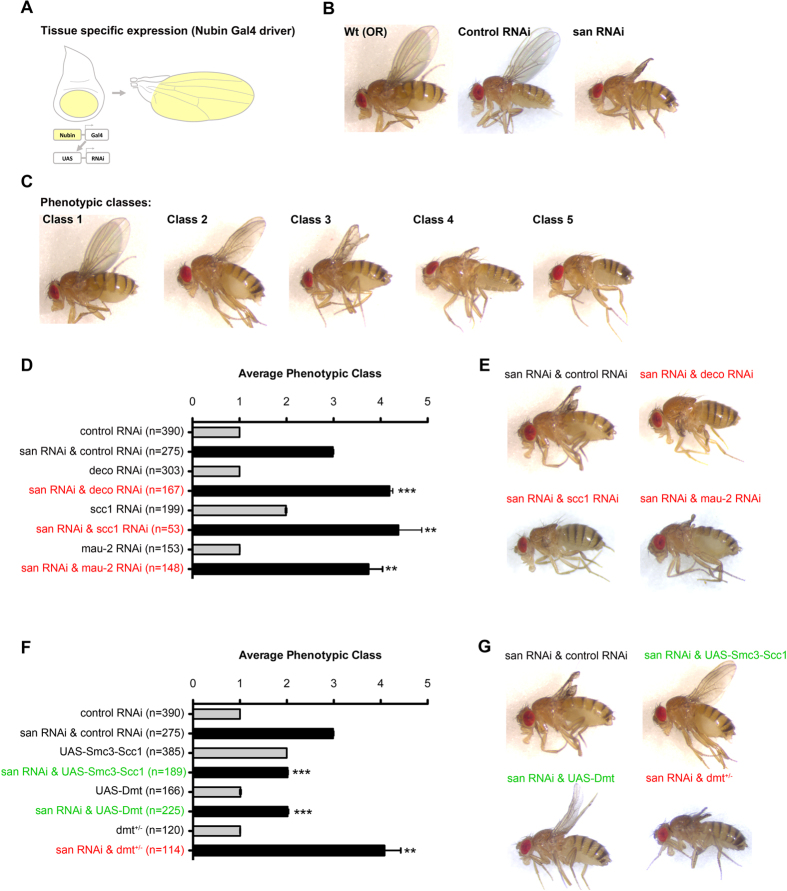
Naa50/San is required for the correct interaction between Scc1 and Smc3. **(A)** Wing blade-specific RNAi using the Nubbin-Gal4 driver^43^,^44^ and the UAS/Gal4 system^42^. **(B)** Adult wings of wild type *Drosophila* (Oregon R; OR), *Drosophila* expressing a control RNAi (*mCherry* RNAi), and *Drosophila* expressing RNAi for *san* in the larvae wing imaginal discs. **(C)** Scored adult wing phenotypic classes: class 1 (wild type wings); class 2 (weak wing phenotype); class 3 (*san* RNAi-like wing phenotype); class 4 (highly abnormal wings); class 5 (absence/vestigial adult wings). **(D)** Quantification of *Drosophila* wing phenotypes expressing individual RNAi transgenes for *mCherry, deco, scc1* or *mau-2* (grey bars) or co-expressing *san* RNAi with *mCherry* RNAi, *deco* RNAi, *scc1* RNAi, or *mau-2* RNAi (black bars) in the larvae wing imaginal discs. **(E)** Representative adult wing phenotype of *Drosophila* co-expressing *san* RNAi with *mCherry* RNAi, *deco* RNAi, *scc1* RNAi or *mau-2* RNAi in the larvae wing imaginal discs. **(F)** Quantification of *Drosophila* wing phenotypes expressing a RNAi transgene for *mCherry*, a *Smc3-Scc1* fusion construct^58^ or overexpressing *Dmt* in the larva wing imaginal discs, and *Drosophila* without one copy of *Dmt (dmt*^+/−^) (grey bars). Quantification of *Drosophila* wing phenotypes co-expressing *san* RNAi with *mCherry*, a *Smc3-Scc1* fusion construct or *Dmt* in the larvae wing imaginal discs, and *Drosophila* without one copy of *Dmt (dmt*^+/−^) expressing *san* RNAi in the larvae wing imaginal discs (black bars). **(G)** Representative adult wing phenotype of *Drosophila* co-expressing *san* RNAi with *mCherry* RNAi, a *Smc3-Scc1* fusion construct or *Dmt* in the larvae wing imaginal discs, and *Drosophila* without one copy of *Dmt (dmt*^+/−^) expressing *san* RNAi in the larvae wing imaginal discs. Phenotypic quantification of adult wings is mean ± S.D. of three independent experiments and is based on the classes described in (**C**) and Supplementary Fig. 1 (**p < 0.01 and ***p < 0.001, Student’s *t* test; *n* represents the total number of flies evaluated). The genotypes are written in green, black, or red if there was, respectively, suppression, no alteration, or enhancement of the original *san* RNAi wing phenotype. Detailed genotypes are indicated in Supplementary Table 1. Top left panels in (**E**) and (**G**), and wild-type panel in (**B**) and Class1 panel in (**C**), are the same.

In order to better understand the mitotic function of Naa50/San during development of multicellular organisms, we performed an enhancer/suppressor screen for genes whose depletion by RNA interference (RNAi) enhanced/suppressed the adult wing phenotypes observed after depletion of Naa50/San (Rui Silva and Rui Gonçalo Martinho, unpublished data). We used a Gal4 driver (Nubbin-Gal4) specific for the larvae imaginal wing discs and capable of inducing transgene expression in the highly proliferative blade region epithelial cells^42^,^43^,^44^ (Fig. 1A). *Drosophila* adult wings showed an abnormal development after depletion of Naa50/San within the larvae imaginal wing discs (expression of *san* RNAi transgene under the control of the Nubbin-Gal4 driver) (Fig. 1B). Co-expression of *san* RNAi and control RNAi transgenes produced adult wing phenotypes identical to the ones previously observed after expression of the *san* RNAi transgene (Fig. 1E, Top left). All scored phenotypic classes are shown in Fig. 1C and Supplementary Fig. 1 (for more experimental detail see methods). In contrast, co-expression of *san* RNAi with distinct RNAi constructs for depletion of the cohesin subunit Scc1^45^,^46^,^47^, of the cohesin loader Mau-2/Scc4^48^, or of the cohesin positive regulator Eco1/Deco acetyltransferase^36^,^49^,^50^, significantly enhanced *Drosophila* adult wing phenotypes (compare black bars in Fig. 1D and wing phenotypes in Fig. 1E). Besides a mild notch-like phenotype in otherwise normal wings after expression of *scc1* RNAi (scored as class 2), none of these enhancer UAS-RNAi constructs with the Nubbin-Gal4 driver produced by themselves significant adult wing phenotypes (Fig. 1D; see grey bars), we concluded the establishment/maintenance of sister chromatid cohesion was most likely particularly important for adult wing development after depletion of Naa50/San.

### Naa50/San is crucial for the correct interaction between Scc1 and Smc3

The cohesin complex has a ring-shaped structure and it is composed of four subunits: Smc1, Smc3, Scc3/SA and Scc1/Rad21/Mcd1^51^,^52^. Coentrapment of sister chromatids occurs during DNA replication. Smc3 is internally acetylated by Eco1 acetyltransferase^49^,^50^ and subsequent Dalmatian/Sororin recruitment^53^ is essential for establishment of stable cohesion as it stabilizes the interaction between the N-terminal domain of Scc1 and the C-terminal domain of Smc3^54^. Eco1 acetylation of cohesin subunit Smc3 promotes cohesion by antagonizing the function of Wapl and/or by altering Smc3 head function^55^,^56^.

Deco, the *Drosophila* ortholog of Eco1, is also important for the establishment of sister chromatid cohesion and normal chromosome segregation during anaphase^36^. Since yeast Wapl mediates the dissociation between Scc1 and Smc3 subunits creating a cohesin’s DNA exit gate^57^, and given the fact that depletion of *Drosophila* Deco specifically enhanced the adult wing phenotypes observed after depletion of Naa50/San (Fig. 1D and E), we hypothesized that Naa50/San function was critical for the interaction between cohesin subunits Smc3 and Scc1. In order to test this hypothesis, we investigated if expression of a fusion construct between Scc1 and Smc3^58^ could suppress the adult wing phenotypes of *san* RNAi-treated wing discs. Consistently, and although expression of this fusion construct by itself weakly impaired wing development (Fig. 1F; see grey bar), its co-expression with *san* RNAi significantly suppressed the wing disc phenotypes observed after depletion of Naa50/San (compare black bars in Fig. 1F and wing phenotypes in Fig. 1G).

Dalmatian/Sororin (Dmt) mediates cohesion by antagonizing Wapl^53^. If Naa50/San function is specifically required for the interaction between Scc1 and Smc3, then overexpression of Dmt should suppress the *san* RNAi adult wing phenotypes. Consistently, whereas overexpression of Dmt significantly suppressed the adult wing phenotypes observed after depletion of Naa50/San (compare black bars in Fig. 1F and wing phenotypes in Fig. 1G), a mutant allele of *dmt* dominantly enhanced *san* RNAi phenotypes (compare black bars in Fig. 1F and wing phenotypes in Fig. 1G). Since Dalmatian/Sororin protein levels are not reduced after depletion of Naa50/San (Fig. 6B) than the observed phenotypes are not due to its destabilization. Altogether, these results suggest that Naa50/San is required for sister chromatid cohesion during *Drosophila* wing development, most likely by regulating (directly or indirectly) the correct interaction between the cohesin subunits Scc1 and Smc3.

### Naa50/San is a positive regulator of sister-chromatid cohesion in *Drosophila* S2 cells

**Figure 2:**
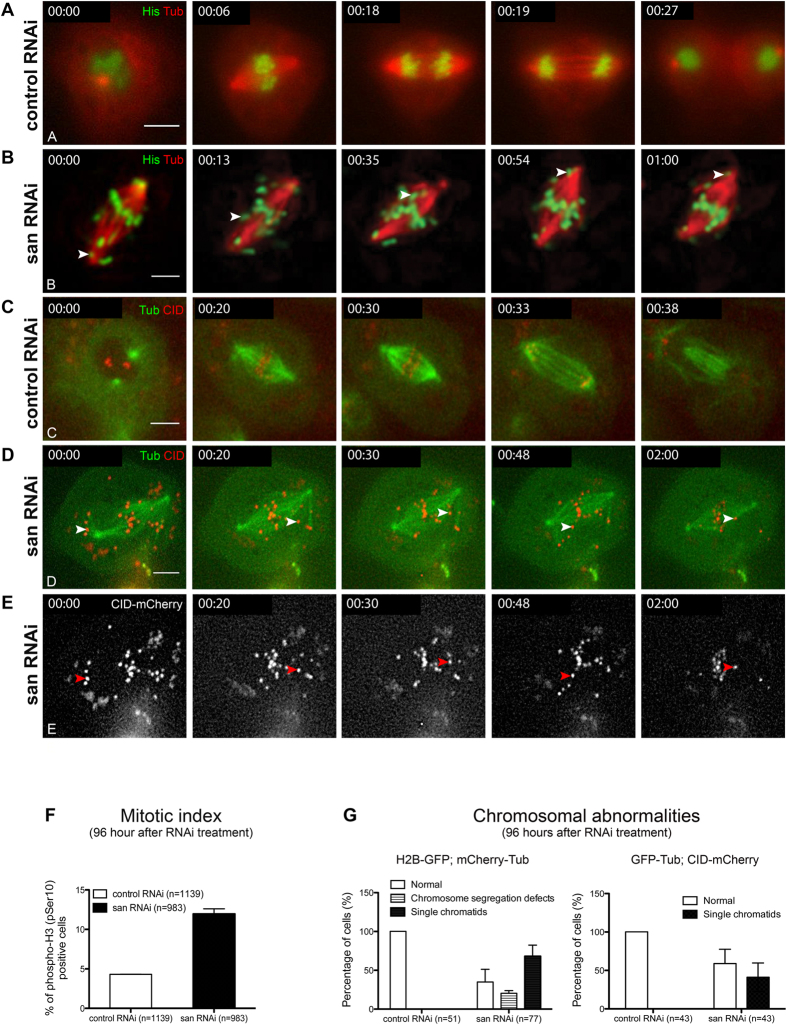
Naa50/San is required for sister chromatid cohesion in *Drosophila* S2 cells. *Drosophila* S2 cells depleted for Naa50/San (*san* RNAi-treated cells) showed sister chromatid cohesion defects (Supplementary Fig. 2A,B and D), with a metaphase arrest, detectable single chromatids during metaphase, and chromosome segregation defects during anaphase. All cells in this figure were analyzed 96 hours after RNAi-treatment. **(A–E)** Selected frames from time-lapse videos (see Movies S1, S2, S5 and S6) of control RNAi and *san* RNAi-treated S2 cells. **(A,C)** Control RNAi-treated cells showed no significant arrest in metaphase or chromosome segregation defects during anaphase (see Movies S1 and S5). **(B,D,E)** *san* RNAi-treated cells show an arrest in metaphase (see Movies S2,S3,S4 and S6) with a significant increase in their mitotic index **(F)**, and chromosome segregation defects during anaphase **(G)**. **(F)** Mitotic index (% of phospho-H3 (pSer10) positive cells) for control RNAi and *san* RNAi-treated cells (96 hours after RNAi-treatment) was, respectively, 4.3% ± 0.2 (n = 1139) and 12.0% ± 0.7 (n = 983) (p < 0.001 Student’s t-test). *san* RNAi but not control RNAi-treated cells showed single chromatids with their typical oscillatory behavior during metaphase (see arrowheads in B,D and E; see Movies S1,S2,S3,S4,S5,S6), which is suggestive of a loss of cohesion. **(G)** Frequency of cells with single chromatids after *san* RNAi-treatment was 5.6% ± 0.1 (n = 17) (72 hours after RNAi-treatment) (see Fig. 3) and 61% ± 11.7 (n = 77) (96 hours after RNAi-treatment). Single chromatids were never detected during mitosis and before anaphase in control RNAi-treated cells. *Drosophila* S2 cells stably expressed GFP-Histone H2B (green) and α-Tubulin-mCherry (red) **(A,B)**^70^ or GFP-α-Tubulin (green) and CID-mCherry (red) **(C–E)**^71^. All images were obtained using maximum intensity projections of Z-stacks (0.8 μm stacks of 5 sections each). For movies, Z-stacks were collected every 30 seconds. Scale bars equal 10 μm. San protein levels after control RNAi and *san* RNAi-treatment are shown in Supplementary Fig. 3A.

**Figure 3:**
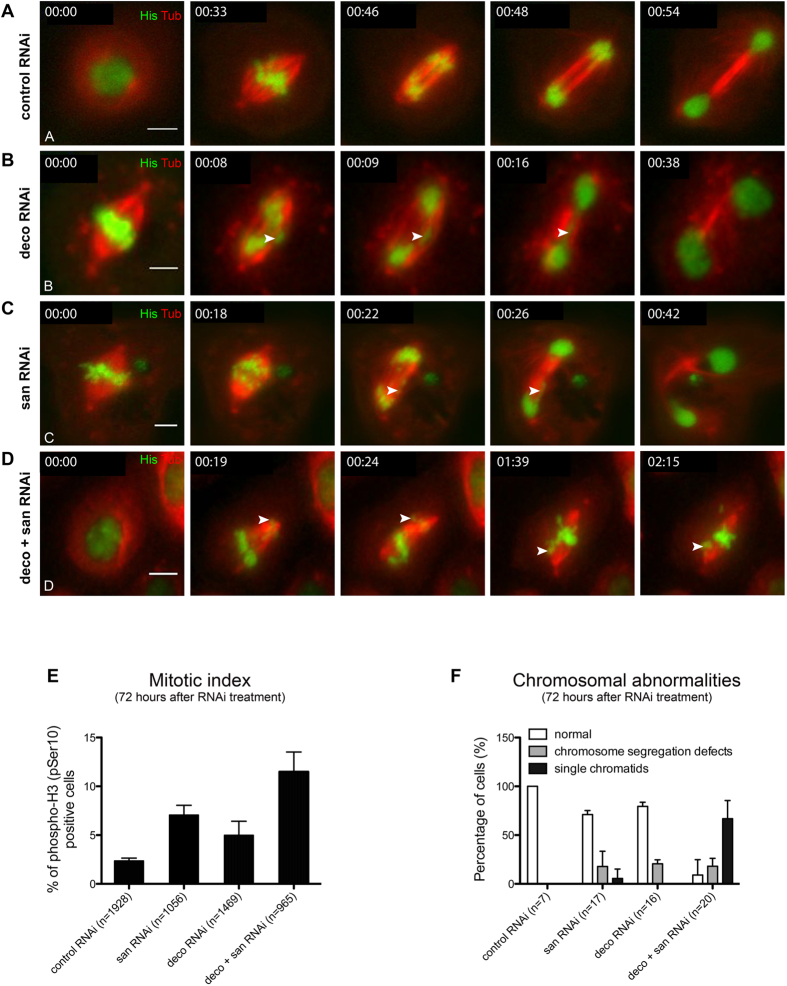
Co-depletion of Deco and Naa50/San significantly enhances *Drosophila* S2 cells chromosome segregation defects. All cells in this figure were analyzed 72 hours after RNAi-treatment. **(A–D)** Selected frames from time-lapse videos (see Movies S7,S8,S9,S10) of control RNAi, *san* RNAi, *deco* RNAi, and *san* RNAi *deco* RNAi co-treated S2 cells (arrowheads indicate single chromatids). *san* RNAi-treated and *deco* RNAi-treated cells showed chromosome segregation defects during anaphase (B,C,F; see arrowheads in B and C) and a significant arrest during mitosis **(E)**, however detectable levels of cells with single chromatids during metaphase were only detected in *san* RNAi-treated cells **(F)**. Co-depletion of Naa50/San and Deco significantly enhanced the mitosis arrest **(E)** and frequency of cells with single chromatids (D,F; see arrowheads in D). **(E)** Mitotic index (% of phospho-H3 (pSer10) positive cells) for control RNAi, *san* RNAi, *deco* RNAi, and *san* RNAi *deco* RNAi co-treated cells (72 hours after RNAi-treatment) was, respectively, 2.4% ± 0.2 (n = 1928), 7.1% ± 0.7 (n = 1056), 5.0% ± 1.0 (n = 1469), and 11.5% ± 1.4 (n = 965). The following mitotic index differences are statistically significant for control RNAi comparing to *san* RNAi treated cells or to *san* RNAi *deco* RNAi co-treated cells; and for *san* RNAi treated cells comparing to *san* RNAi *deco* RNAi co-treated cells (p < 0.05 Student’s t-test). **(F)** Frequency of cells with chromosome segregation defects during anaphase after control RNAi, *san* RNAi, *deco* RNAi, and *san* RNAi *deco* RNAi co-treated cells (72 hours after RNAi-treatment) was, respectively, none (0%) (n = 7), 18% ± 0.1 (n = 17), 20% ± 0.1 (n = 16), and 18% ± 0.1 (n = 20). **(F)** Frequency of cells with single chromatids after control RNAi, *san* RNAi, *deco* RNAi, and san RNAi *deco* RNAi co-treated cells (72 hours after RNAi-treatment) was, respectively, none (0%) (n = 7), 5.6% ± 0.1 (n = 17), none (0%) (n = 16), and 66.8% ± 0.2 (n = 20). The difference between frequency of cells with single chromatids in *san* RNAi treated cells and *san* RNAi *deco* RNAi co-treated cells is statistically significant (p < 0.01 Student’s t-test). *Drosophila* S2 cells stably expressed GFP-Histone H2B (green) and α-Tubulin-mCherry (red) **(A–D)**^70^. All images were obtained using maximum intensity projections of Z-stacks (0.8 μm stacks of 5 sections each). Scale bars equal 10 μm.

In order to better understand the function of Naa50/San in sister-chromatid cohesion, we performed live-cell imaging of cells depleted for Naa50/San. Since L3 larvae neuroblasts mutant for *san* (zygotic mutants) were highly abnormal, with an extremely low mitotic index^36^ and a highly aberrant ploidy (data not shown), tissue culture *Drosophila* Schneider 2 (S2) cells were used instead to study the mitotic function of Naa50/San. As expected, Naa50/San was important for centromeric sister chromatid cohesion in *Drosophila* S2 cells; its depletion by RNAi was associated with an abnormal association of Scc1 to chromatin (Supplementary Fig. 2A,B and D), an increase in the cells mitotic index (Fig. 2F), and an abnormal segregation of chromosomes during anaphase (Figs 2G and 3C). Interestingly, Naa50/San-depleted S2 cells also showed a significant amount of single chromatids (see arrowheads in Fig. 2B,D and E; see quantification in Fig. 2G; Movies S1,S2,S3,S4,S5,S6; see arrowheads in Movies S2,S3,S4, and S6) that was suggestive of a general loss of sister chromatid cohesion after depletion of Naa50/San. The single chromatids showed the typical oscillatory behavior where they moved towards and away from the spindle poles as they were captured by microtubules and their association to their unique kinetochore was subsequently destabilized by Aurora B^59^,^60^,^61^,^62^,^63^. Identical results were obtained in *Drosophila* S2 cells with a distinct non-overlapping double-stranded RNA against *san* (Movie S4).

In order to investigate if, similarly to the adult wing phenotypes (Fig. 1D and E), depletion of Deco could enhance the mitotic phenotypes of *san* RNAi-treated S2 cells, we analyzed the mitotic defects of S2 cells 72 hours after RNAi-treatment (lower depletion of Naa50/San when compared to 96 hours after RNAi-treatment (Supplementary Fig. 3A)). Simultaneous co-depletion of Naa50/San and Deco (*san* RNAi and *deco* RNAi) significantly increased the number of cells with detectable single-chromatids (see arrowhead in Fig. 3D; see quantification in Fig. 3F; Movies S9 and 10; see arrowheads in Movie S10) and their mitotic index (Fig. 3E) when compared to control and single RNAi-treated cells (Fig. 3A–D; see quantification in Fig. 3F). Since an extended arrest in metaphase with bi-oriented chromosomes can potentially be associated with partial loss of cohesion^64^,^65^, we investigated if single chromatids could be detected immediately after metaphase arrest. Live-cell imaging of cells depleted for Naa50/San and co-depleted for Naa50/San and Deco showed detectable single chromatids immediately after metaphase arrest (see arrowhead in Movie S3 and S10). This suggested that the observed single chromatids resulted from *bona fide* cohesion defects, and were not simply due to the extended metaphase arrest. Altogether, these results suggest that Naa50/San is a positive regulator of sister chromatid cohesion, possibly by facilitating (directly or indirectly) the interaction between the cohesin subunits Scc1 and Smc3.

### Naa50/San catalytic activity is required for chromosome segregation during mitosis

**Figure 4:**
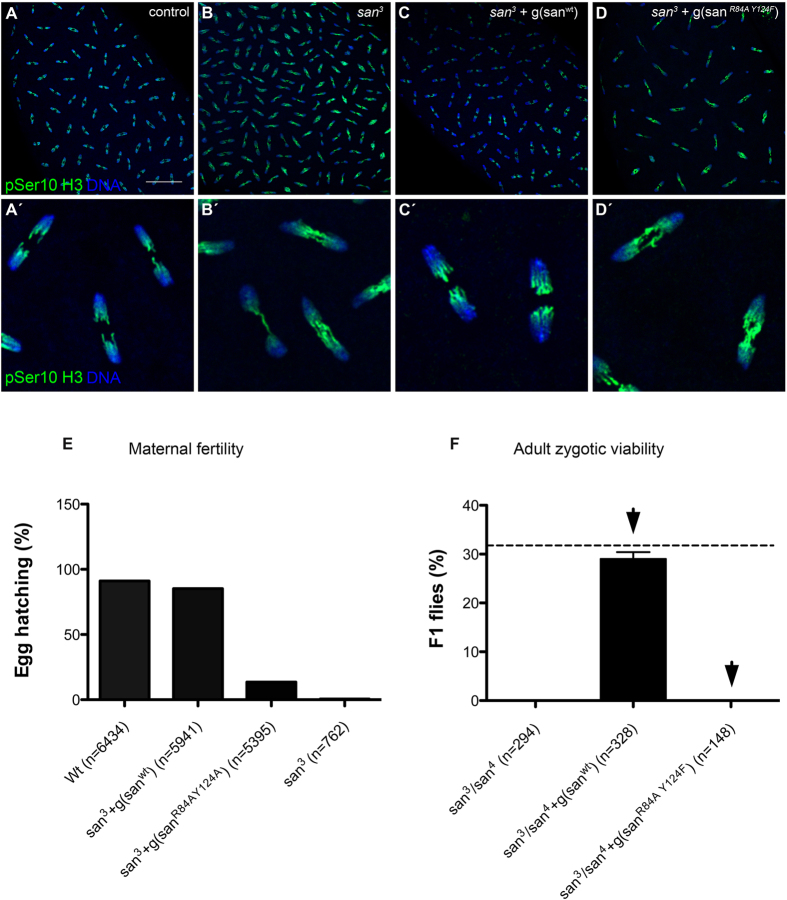
The catalytic activity of Naa50/San is essential for viability and normal mitosis. A wild type (g(*san*^*wt*^)), but not a catalytically dead-version (g(*san*^*R84A Y124F*^)) of a genomic construct that encodes Naa50/San, was able to rescue the chromosome segregation defects of syncytial blastoderm embryos mutant for *san*^*3*^ (maternal mutants)^40^ **(A–D’)**, the fertility of females whose germ line was mutant for *san* **(E)**, and the adult viability of zygotic mutants of *san* **(F)**. Loss of Naa50/San (San^R84A Y124F^) mutant protein Nt-Ac catalytic activity is shown in Fig. 5B. (**A**–**D’**) All panels show syncytial blastoderm embryos. Control wild type (**A**,**A’**), *san* mutant (*san*^*3*^) (**B**,**B’**), *san* mutant with a wild type genomic construct (*san*^*3*^ + g(*san*^*wt*^)) (**C,C’**), and *san* mutant with a catalytically dead genomic construct (*san*^*3*^ + g(*san*^*R84A Y124F*^)) (**D,D’**). Both wild type (g(*san*^*wt*^)) and catalytically dead genomic constructs (g(*san*^*R84A Y124F*^)) contained the gene endogenous minimal promoter and were integrated in the same attP2 site. All embryos were stained for DNA (blue) and pSer10 Histone H3 (green). **(E)** Embryonic hatching of fertilized eggs laid by females whose germ line was wild type (control), mutant for *san* with a wild type genomic construct (*san*^*3*^ + g(*san*^*wt*^)), mutant for *san* with a catalytically dead genomic construct (*san*^*3*^ + g(*san*^*R84A Y124F*^)), and mutant for *san (san*^*3*^) was, respectively, 91.1% ± 5.6 (n = 6434), 85.1% ± 1.6 (n = 5941), 13.5% ± 2.4 (n = 5395), and 0.5% ± 0.7 (n = 762). **(F)** A genomic construct carrying a wild type copy of *san* efficiently rescued the zygotic lethality of two loss-of-function alleles of *san*^40^ (adult viability: *san* mutant [*san*^*3*^/*san*^*4*^] = 0% (n = 294, adult flies); *san* mutant + g(*san*^*wt*^) [*san*^*3*^/*san*^*4*^; g(*san*^*wt*^)/+] = 29% ± 1 (n = 328, adult flies; full complementation should correspond to 33% of total *Drosophila* flies (dashed line)). A genomic construct carrying a catalytically dead allele of *san* g(*san*^*R84A Y124F*^) failed to rescue the zygotic lethality of two loss-of-function alleles of *san* (adult viability: *san* mutant [*san*^*3*^/*san*^*4*^] = 0% (n = 294, adult flies); *san* mutant + g(*san*^*R84A Y124F*^) [*san*^*3*^/*san*^*4*^; g(*san*^*R84A Y124F*^)/+] = 0% (n = 148, adult flies; full complementation should correspond to 33% of total *Drosophila* flies). For more experimental detail see Supplementary Table 3.

**Figure 5:**
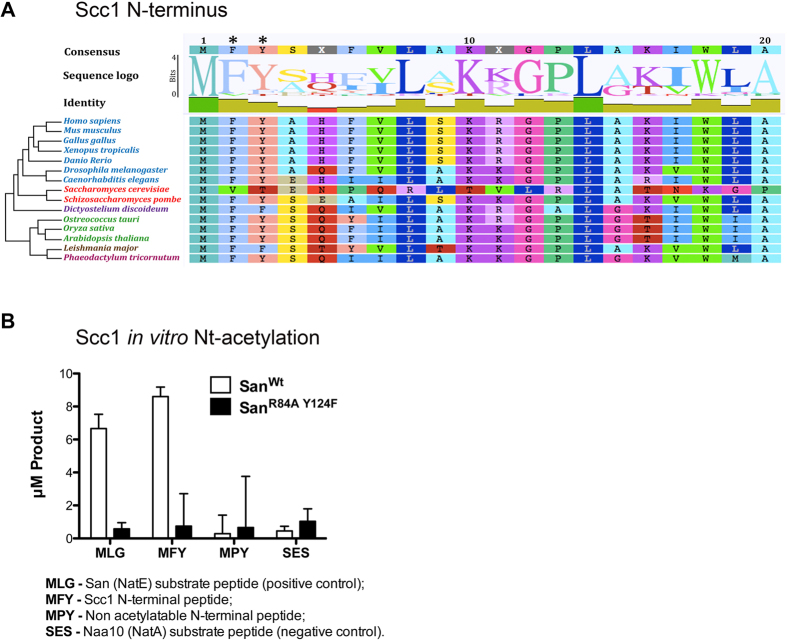
Naa50/San N-terminally acetylates Scc1. The N-terminal phenylalanine and tyrosine residues of Scc1 are highly conserved. The Scc1 N-terminal sequence (MFY-) is efficiently Nt-acetylated *in vitro* by Naa50/San. **(A)** Orthologs of Scc1 from 15 distinct species representative of the eukaryotic tree of life were retrieved using reciprocal bidirectional protein BLAST analysis. N-terminal sequences were aligned using the Geneious R7 software. The N-terminal second residue phenylalanine (F) and third residue tyrosine (Y) are highly conserved across the eukaryotic tree of life (see asterisks). The height of the letters represents the overall relative degree of residue conservation. **(B)** Wild-type *Drosophila* Naa50/San efficiently N-terminally acetylated *in vitro* Scc1 N-terminal peptide (MFY-) and a previously defined Naa50/San substrate MLG- peptide (positive control). In contrast, Naa50/San did not efficiently N-terminally acetylate a Naa10 substrate peptide SES- (negative control), nor the non-acetylatable N-terminal peptide MPY-. A catalytically dead-version of Naa50/San (San^R84A Y124F^) failed to efficiently N-terminally acetylate all tested peptides. Both wild type Naa50/San and Naa50/San^R84A Y124F^ were heterologously expressed in *E. coli* (see methods). All measurements were done in the linear range of enzymatic activity (data not shown).

Previously it was reported that reduction of human Naa50/San catalytic activity impaired its mitotic function^39^. Yet, since the reported mutant protein had a significant amount of residual catalytic activity and it was not expressed at endogenous levels, it was still unclear to what extent the mitotic function of Naa50/San relied on its catalytic activity. Since Naa50/San interacts with NatA^36^,^38^ and its loss may partially impair the catalytic activity of NatA^19^,^35^, Naa50/San can potentially have functions independent of its catalytic activity. In order to investigate if Naa50/San catalytic activity is required for normal chromosome segregation during mitosis, we generated a transgenic genomic construct of the *san* gene locus, under the control of its own promoter for endogenous protein expression levels (Supplementary Fig. 3B), and carrying two different amino acid mutations (R84A Y124F) that completely abrogated the *in vitro* catalytic activity of this enzyme (Fig. 5B). Consistent with the hypothesis that the catalytic activity of Naa50/San is essential for sister chromatid cohesion and normal mitosis, a wild-type genomic construct (g(*san*^*Wt*^)), but not the catalytically dead genomic construct of *san* (g(*san*^*R84A Y124F*^)), fully rescued the lethality of zygotic loss-of-function mutant alleles of *san* (Fig. 4F; Supplementary Table 3), the sterility of females whose germ-line was mutant for *san* (germ-line clones) (Fig. 4E), and the mitotic defects of syncytial blastoderm embryos mutant for *san* (maternal mutants) (Fig. 4A–D’)^36^,^40^. These results fully support the hypothesis that Naa50/San catalytic activity is essential for chromosome segregation during mitosis.

### Naa50/San N-terminally acetylates Scc1

NATs have distinct substrate specificities and their substrate recognition during protein translation depends on the nature of the first 2–5 amino acids of the elongating substrate polypeptide^7^,^10^,^31^,^32^,^33^. Analysis of the N-termini of all cohesin subunits showed that the N-terminal second and third amino acid residues of Scc1 were highly conserved across the eukaryotic tree of life (Fig. 5A). Since the Scc1 N-termini (MFY-) is compatible with the substrate specificity of Naa50/San^21^,^35^, we hypothesized that Naa50/San N-terminally acetylates Scc1. In contrast, the N-terminal sequences of the other cohesion subunits were less conserved and/or did not match the substrate specificity of Naa50/San (data not shown). In order to test this hypothesis we investigated if *Drosophila* Naa50/San was able to specifically N-terminally acetylate an Scc1 N-terminal peptide *in vitro*. We observed that the wild-type Naa50/San (San^Wt^), but not the catalytically dead mutant (San^R84A Y124F^), efficiently N-terminally acetylated a known substrate peptide of Naa50/San (positive control) and Scc1 N-terminal peptide (Fig. 5B). In contrast, Nt-Ac of a NatA substrate peptide and a Scc1 N-terminal peptide with a proline mutation that is known to block Nt-Ac^9^ (negative controls) was poor (Fig. 5B). Multiple attempts to confirm that Scc1 Nt-Ac was also reduced *in vivo* after depletion of Naa50/San were inconclusive, as the N-terminal peptide of Scc1 was not detected by mass spectrometry (data not shown).

### Naa50/San is not required for overall integrity of the cohesin complex

Although the steady-state protein levels of Scc1 and Smc1 were normal in cells depleted for Naa50/San (Supplementary Fig. 2C), Scc1 association to the mitotic metaphase chromosomes was nevertheless impaired (Supplementary Fig. 2A,B and D)^36^,^39^.

**Figure 6:**
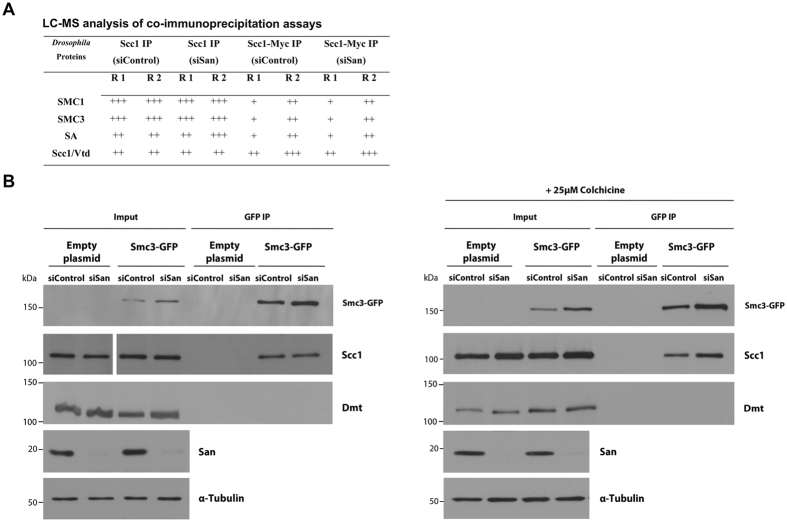
Naa50/San is not required for overall integrity of the cohesin complex. **(A)** All subunits of the cohesin complex, but not Dalmatian/Sororin, were efficiently immunoprecipitated with endogenous Scc1 or with Myc-tagged Scc1 after depletion of Naa50/San. Co-immunoprecipitations with an anti-Scc1 antibody or with anti-c-Myc Magnetic beads (Invitrogen, Grand Island, NY, USA) were performed, respectively, using total protein extracts from *Drosophila* S2 cells or from S2 cells expressing a Myc-tagged Scc1. Both sets of cells were either treated with control RNAi or *san* RNAi before immunoprecipitation. (−), (+), (++), and (+++) corresponds, respectively, to 0, 1–9, 10–19, and >20 non-repeated peptides detected by LC-MS. R1 and R2 correspond to replica 1 and replica 2, respectively. None of the proteins shown in this analysis were detected in the negative controls (respectively, pre-immune serum or *Drosophila* S2 cells expressing an empty plasmid). For detailed LC-MS analysis see Supplementary Table 4. **(B,C)** Endogenous Scc1 was efficiently co-immunoprecipitated by Smc3-GFP after depletion of Naa50/San, both in actively dividing **(B)** or metaphase-arrested **(C)** cells. On the other hand, Dalmatian/Sororin (Dmt) was not co-immnunoprecipitated by Smc3-GFP in both conditions. Co-immunoprecipitation from total protein extracts from *Drosophila* S2 cells expressing GFP-tagged Smc3 (Smc3-GFP) and using anti-GFP coated Dynabeads. Protein extracts from *Drosophila* S2 cells transfected with an empty plasmid were used as a negative control. For metaphase arrest, *Drosophila* S2 cells were treated with 25 μM of colchicine for 12 hours. The mitotic index (% of phospho-H3 (pSer10) positive cells) of S2 cells treated or not with colchicine is shown in Supplementary Fig. 6.

In order to investigate if depletion of Naa50/San impaired the overall integrity of the cohesin complex, endogenous Scc1 or a Myc-tagged Scc1 fusion protein expressed in *Drosophila* S2 cells were immunoprecipitated from actively dividing cells. All subunits of the cohesin complex, but significantly not Dalmatian/Sororin, were efficiently immunoprecipitated in both cases from total protein extracts prepared from control and from Naa50/San-depleted cells (Fig. 6A; detailed results are shown in Supplementary Table 4), suggesting that the overall integrity of the cohesin complex was not impaired after depletion of Naa50/San. Differences in the interaction between Scc1 and Smc3 in dividing or metaphase-arrested cells was similarly not detectably affected after depletion of Naa50/San from total protein extracts (Fig. 6B,C). Since Dalmatian/Sororin was not immunoprecipitated with endogenous Scc1, Myc-tagged Scc1 (Fig. 6A) or GFP-tagged Smc3 (Fig. 6B,C), most of the isolated cohesin complex was most likely not interacting with sister chromatids in a fully cohesive-state. These results suggest that although depletion of Naa50/San impairs the correct establishment/ maintenance of cohesion, differences in interaction between Scc1 and Smc3 are not detectable by co-immunoprecipitation possibly because they were bridged together by Smc1.

### Ectopic expression of Scc1 suppress the mitotic defects observed after depletion of Naa50/San

**Figure 7:**
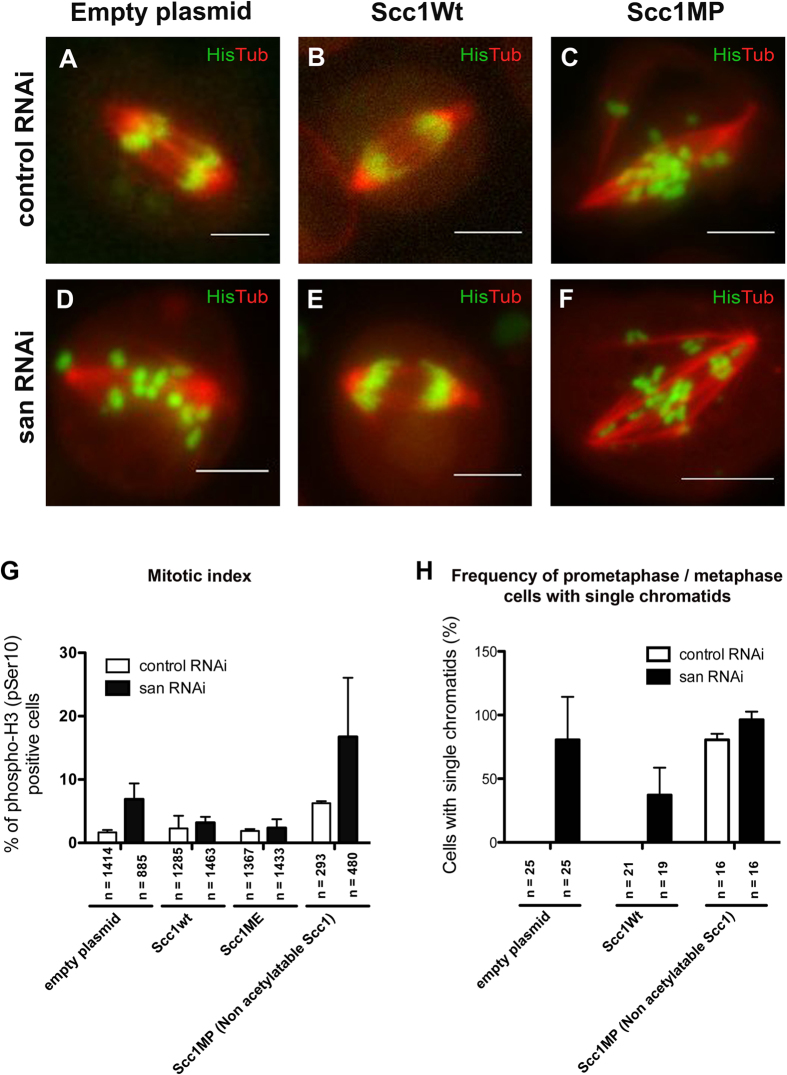
Ectopic expression of Scc1 suppress the mitotic defects observed after depletion of Naa50/San. All cells in this figure were analyzed for 96 hours after RNAi-treatment. **(A–F)** Selected frames from time-lapse videos (see Movies S11,S12,S13,S14,S15,S16) of control RNAi and *san* RNAi-treated S2 cells that were transiently expressing an empty plasmid, wild type Scc1, or mutant Scc1 (Scc1MP). **(G)** Mitotic index of control RNAi and *san* RNAi-treated cells, when carrying an empty plasmid (pHW) was respectively 1.7% ± 0.3 (n = 1414) and 6.9% ± 2.0 (n = 885); when transiently expressing wild type Scc1 (Scc1Wt) it was, respectively, 2.3% ± 1.6 (n = 1285) and 3.2% ± 0.7 (n = 1463); when transiently expressing the mutant variant Scc1ME it was, respectively, 1.9% ± 0.2 (n = 1367) and 2.4% ± 1.1 (n = 1433); when transiently expressing mutant variant Scc1MP it was, respectively, 6.2% ± 0.3 (n = 293) and 16.7% ± 9.3 (n = 480). The following mitotic index differences are statistically significant: for control RNAi comparing to *san* RNAi treated cells, both carrying an empty plasmid; *san* RNAi treated cells with an empty plasmid comparing to *san* RNAi treated cells transiently expressing wild type Scc1 or the mutant variant Scc1ME (p < 0.05 Student’s t-test). **(H)** The frequency of metaphase cells with single chromatids in control RNAi and *san* RNAi-treated cells, when carrying an empty plasmid (pHW), was respectively, 0% (n = 25) and 85.0% ± 29.1 (n = 25); when transiently expressing wild type Scc1 (Scc1Wt) it was, respectively, 0% (n = 21) and 37.1% ± 21.5 (n = 19); when transiently expressing mutant variant Scc1MP it was, respectively, 80.6% ± 4.8 (n = 16) and 96.3% ± 6.4 (n = 16). The following differences in the frequency of metaphase cells with single chromatids are statistically significant: for control RNAi comparing to san RNAi-treated cells, both carrying an empty plasmid (pHW), a wild-type Scc1 (Scc1Wt), or the mutant variant Scc1MP; for *san* RNAi treated cells with an empty plasmid, comparing to *san* RNAi treated cells transiently expressing wild type Scc1 (Scc1Wt) (p < 0.05 Student’s t-test). *Drosophila* S2 cells stably expressed GFP-Histone H2B (green) and α-Tubulin-mCherry (red) **(A–F)**^70^. All images were obtained using maximum intensity projections of Z-stacks (0.8 μm stacks of 5 sections each). Scale bars equals 10 μm.

If Scc1 was truly rate limiting for sister chromatid cohesion in Naa50/San-depleted cells (Supplementary Fig. 3A), then ectopic Scc1 expression should be able to suppress their mitotic defects. Consistently, we observed that ectopic expression of wild type Scc1 significantly suppressed the chromosome segregation defects (Fig. 7A,B,D and E), the mitotic arrest (mitotic index) (Fig. 7G), and the frequency of cells with single chromatids (Fig. 7D,E; see quantification in Fig. 7H; see Movies S11,S12,S13,S14) observed after depletion of Naa50/San. The fact that this suppression occurred without a major increase in the total steady state levels of Scc1 (Supplementary Fig. 3C) also suggested that these cells are extremely sensitive to small changes of Scc1.

The N-terminal second and third amino acid residues of Scc1 (MFY-) are highly conserved (Fig. 5A), and they are compatible with Naa50/San substrate specificity^21^,^35^. If Nt-Ac of Scc1 were one of the main reasons why Scc1 N-terminus is conserved, then a mutation capable of ensuring efficient Nt-Ac should only have a limited impact on Scc1 function. Consistently, ectopic expression of a mutant Scc1, where the second residue was changed to a Glutamate residue (from MF- to ME-; a NatB substrate likely to be fully Nt-Ac *in vivo*) (Supplementary Fig. 3D), was still able to significantly suppress the mitotic arrest after depletion of Naa50/San (Fig. 7G). In contrast, ectopic expression of a mutant Scc1 protein whose N-terminal second residue was mutated to a Proline residue (Supplementary Fig. 3D) (MF- to MP-; resulting in a proline N-terminus after methionine cleavage, which completely blocks protein Nt-Ac^9^), failed to suppress the mitotic arrest and the single chromatids observed after depletion of Naa50/San (Fig. 7D and F; see quantification in Fig. 7G and H; see Movie S16; for Scc1 protein expression see Supplementary Fig. 3C).

Ectopic expression of Scc1 (MP-) mutant protein induced, with or without depletion of Naa50/San, a major arrest in mitosis (Fig. 7G) and significantly increased the number of cells with loss of cohesion and single chromatids (Fig. 7A,C,D and F; see quantification in Fig. 7H; see Movies S15 and S16). The unexpected dominant negative effect of Scc1 (MP-) might result not only due to a complete blockage of Scc1 Nt-Ac but also from the conformational rigidity of proline and/or the expected loss of the Scc1 initiator methionine^66^. Either way, both possibilities highlight the functional importance of the first two N-terminal amino acids of Scc1 in the establishment of a stable interaction with Smc3 and for normal segregation of chromosomes during mitosis.

## Discussion

We propose that in higher eukaryotes Naa50/San is required for mitotic sister chromatid cohesion by facilitating the correct interaction between Scc1 and Smc3. Yet, and in contrast with the nuclear Eco1/Deco lysine acetyltransferase whose role in Smc3 internal acetylation is important for the establishment of sister chromatid cohesion during DNA replication^36^,^49^,^50^, our results suggest that Naa50/San is most likely required but not instructive for the establishment/maintenance of cohesion. Although depletion of Naa60 (NatF) in *Drosophila* S2 cells results in the appearance of mitotic phenotypes^10^, adult *Drosophila* flies deleted for Naa60 are nevertheless viable (Rui Gonçalo Martinho, unpublished data) suggesting that Naa50/San is the dominant mitotic NAT. The *in vitro* substrate specificities of Naa50 and Naa60 are overlapping^10^,^33^, but these two enzymes appear to have non-overlapping *in vivo* substrates^67^.

Naa50/San is in all likelihood not directly involved in the establishment of cohesion during DNA replication since Nt-Ac usually occurs during protein translation^37^,^68^ and, contrary to Eco1 acetyltransferase, Naa50/San is not enriched in the nucleus (Supplementary Fig. 4)^36^,^67^. Furthermore, and although human Naa50/San can potentially internally acetylate beta-Tubulin *in vivo*^41^, the kinetics of this reaction is most likely highly unfavourable as Naa50/San catalytic site cannot easily accommodate a side-chain lysine substrate^34^.

Naa50/San N-terminal acetylates Scc1 *in vitro* and most likely *in vivo*. Although the precise structural consequences of Scc1 Nt-Ac are still unknown and we failed to demonstrate that Scc1 is an *in vivo* substrate of Naa50/San, we propose that Naa50/San N-terminally acetylates the nascent Scc1 polypeptide during translation, which is potentially important for the correct folding of Scc1 N-terminal domain, and its subsequent interaction with Smc3 during the establishment of sister chromatid cohesion. Consistently, Scc1 N-terminus is highly conserved, its sequence is consistent with it being an *in vivo* substrate Naa50/San, and a mutation that blocks Nt-Ac of Scc1 impairs its function.

Why Naa50/San is not required for female germ line stem cell mitotic divisions is still unclear^40^, but it raises the possibility that the requirement of this enzyme varies during development, possibly due to redundancy with other NATs and/or tissue-specific regulators of sister chromatid cohesion. Alternatively, it is also possible that Nt-Ac of Scc1 is not always required for cohesion and Scc1 function. This is consistent with the observation that whereas *Drosophila* and human Naa50/San are required for sister chromatid cohesion, their orthologs in budding yeast are not^37^,^39^. Future work will clarify the molecular nature of the differential requirements of Naa50/San during *Drosophila* development.

## Material and Methods

### Fly work and genetics

Flies were raised using standard techniques. All *Drosophila* stocks used in this study are listed in Supplementary Table 1. The *san* alleles were isolated in a previously reported study^40^. Maternal mutant embryos and germ-line mutant clones were generated using the FLP/FRT *ovoD* system^69^. Germ-line clones of *san*^*3*^ and *san*^*4*^ were established by crossing FRT42B *san*/CyO virgins to *hs-*FLP; FRT42B *ovo*^*D*^/CyO males. The progeny was heat shocked twice at 37 °C for 90 min during second and third larval instar stages. As control we generated germ-line clones with FRT42B by crossing FRT42B/CyO virgins to *hs-*FLP; FRT42B *ovo*^*D*^/CyO males, followed by the heat shock procedure described before.

*san* loss-of-function mutant alleles^40^ were complemented with a transgene carrying a genomic construct that contained a wild-type copy of the *separation anxiety (san*) gene locus with its own endogenous promoter (*san*^*wt*^). To check the requirement for Naa50/San catalytic activity, an identical genomic construct with two different amino acid mutations that rendered Naa50/San enzyme catalytically inactive (*san*^*R84A Y124F*^) was also generated. Both constructs were similarly integrated in a genomic attP2 site. w; FRT42B, *san*^*3*^/CyO virgins were crossed to w; FRT42B, *san*^*4*^/CyO; san^wt^/TM6b males or to w; FRT42B, *san*^*4*^/CyO; *san*^*R84A,Y124F*^/TM6b males. Reciprocal crosses were also performed. Offspring were counted to determine viability of zygotic *san* mutant rescued by the two distinct genomic constructs. Maternal phenotypes were also analyzed. Embryos laid by w; FRT42B, *san*^*3*^/FRT42B *san*^*4*^; san^wt^/+ females or by w; FRT42B, *san*^*3*^/FRT42B *san*^*4*^; *san*^*R84A Y124F*^/+ were fixed and their early embryonic mitotic phenotypes analyzed. Complementation of *san*^*3*^ germ-line clones (see above)^40^,^69^ with *san*^*wt*^ *and san*^*R84A Y124F*^ genomic constructs was also performed. w; FRT42B *san*^*3*^/CyO; san^wt^*/TM6B and w;* FRT42B *san*^*3*^/CyO; *san*^*R84A Y124F*^/TM6B virgins were crossed with *hs-*FLP; FRT42B *ovo*^*D*^/CyO males, followed by the heat shock procedure as described above.

### Genetic interaction studies: adult wings

The Gal4/UAS system^42^ was used with the nubbin-Gal4 driver for tissue-specific expression in the blade regions of the larvae wing disc^43^,^44^. For adult wings genetic interactions studies five virgins w; nubbin-GAL4 UAS-san RNAi/CyO or five virgins w; nubbin-Gal4 were mated with 5–7 males from fly stocks containing distinct UAS-RNAi constructs or mutant alleles for genes of interest. The non-CyO progeny was scored into five distinct phenotypic classes accordingly to adult wings abnormalities (Fig. 1C). The crosses were repeated three times and the phenotypic class of more than thirty flies was evaluated for each cross.

### Cell culture of *Drosophila* S2 cells

*Drosophila* Schneider 2 (S2) cells were maintained in Schneider’s *Drosophila* complete medium: Schneider’s insect medium (Sigma), supplemented with 1x L-glutamine, 1x PenStrep, and 10% Fetal bovine serum (Invitrogen) at 25 °C.

### Double-stranded RNA interference of *Drosophila* S2 cells

S2 cells were cultured at 25 °C and RNAi was performed according to standard procedures. To deplete Naa50/San (encoded by *san*/*CG12352*) or Deco (encoded by *deco*/*CG8598*), S2 cells were transfected with double stranded RNAs (dsRNAs) corresponding to approximately 300–400 base pair fragments of each gene. To simultaneously deplete Naa50/San and Deco, S2 cells were simultaneously transfected with both dsRNAs. dsRNAs for GFP was used as control (sequence of used primers is shown in Supplementary Table 2). Each primer incorporates a T7 RNA polymerase-binding site. PCR products were used as template to synthesize dsRNA using the T7 RiboMAX Express kit (Promega). *Drosophila* S2 cells were counted and diluted to 2.5 × 10^6^ cells/ml in serum free medium (SFM) supplemented with L-glutamine. Cells were incubated during 1 hour with 40 μg for each dsRNA at a concentration of 10 μg/ml. After 1 hour incubation with dsRNA, 3 ml of complete media was added back. S2 cells grew in the presence of the diluted dsRNAs and were analyzed 72 hours (Fig. 3) and 96 hours (Fig. 2) after dsRNA treatment. The double amount of control dsRNA was used in Fig. 3 to control for the total amount of dsRNAs used in the co-treatment with san and deco dsRNAs.

### Live-cell imaging of *Drosophila* S2 cells

Live-cell imaging of S2 cells was done using S2 cells stably expressing GFP-Histone H2B and mCherry-α-tubulin^70^ or GFP-α-tubulin and mCherry-centromere identifier (CID, a kinetochore marker)^71^ (kindly provided by Helder Maiato (IBMC, Portugal)). The cells were cultured for 72 hours (in Fig. 3) or 96 hours (in Fig. 2) after RNAi-treatment, as described above. Cells were resuspended and plated in MatTek plates (P35G-1.5–20 C) pre-coated with Concanavalin-A 0.25 mg/ml (C2010; Sigma) 2 hours before observation.

Visualization of live cells was performed using a Delta Vision Core System (Applied Precision) using a 100x UplanSApo objective and a cascade 1 K EMCCD camera (Photometrics). Images were acquired for a period of 2 hours at a frame capture rate of one every 30 seconds and a series of z-sections separated by 0.8-μm intervals using softWoRx (Applied Precision, Inc.). Deconvolution was performed using the conservative ratio method in softWoRx software. Image sequences were converted to movies using the program ImageJ (http://rsb.info.nih.gov/ij/).

### Transfection protocol of *Drosophila* S2 cells

S2 cells transfection with *scc1* transgenic constructs was done using FuGENE^®^ HD Transfection Reagent (E2311) (Promega). Cells were transfected using the reverse transfection protocol. A mix of 100 μL of Serum Free Medium (SFM), 400 ng of DNA, and 4 μL of Fugene HD was incubated at room temperature for 15 min. Meanwhile, cells were plated into 6 well plates at a concentration of 2.5 × 10^6^ cells/well in serum free medium. The mix was added to the cells. After 4 h incubation at 25 °C, complete medium (with 10% Fetal bovine Serum) was added to stop transfection. Cells were incubated for 48 hours at 25 °C before starting the double-stranded RNA interference experiments (described above).

### Generation of constructs and cloning

*Drosophila Scc1* open-reading frame (ORF) was obtained from a full-length *Scc1* cDNA (clone FI11703). Wild type and mutant *Scc1* ORFs where the second N-terminal amino acid residue was mutated to a Glutamate or Proline were cloned into pDONR221 (gateway system, Invitrogen). The N-terminal second residue point mutations were performed using the primers described in Supplementary Table 2. The wild type and mutant *scc1* (known in *Drosophila* as *verthandi*) open reading frames were subcloned into a pHW vector with the *Hsp70* promoter (gateway system, Invitrogen).

### Immunofluorescence microscopy

#### Drosophila S2 cells

After treatment with dsRNAs (see above), 2 × 10^6^ cells were added to coverslips by 1 hour incubation at 25 °C. Cells were fixed with 4% formaldehyde, 0.03 M PIPES, 0.11 M HEPES, 0.01 M EGTA and 4 mM MgSO_4_ for 10 min, followed by two washes in 1x PBS. Permeabilization and blocking was performed for 1 hour with PBS-TB (PBS, 0.1% Triton X-100, 1% Albumin from bovine serum). Primary antibody incubations were done in blocking solution for 2 hours at room temperature or overnight at 4 °C, followed by three 5 min washes in PBS-TB. Secondary antibody incubations were performed as described for the primary antibodies, including three 5 min washes. Primary antibodies included mouse anti-α-tubulin (DM1A) at 1:500 (Sigma), rabbit anti-pSer10-Histone H3 at 1:500 (Upstate Biotechnology), rabbit anti-Scc1 at 1:2000^72^ (kindly provided by Claudio Sunkel (IBMC, Portugal)). Secondary antibodies used were anti-mouse Alexa Fluor 488 and anti-rabbit Alexa Fluor 555 at 1:1000 (Jackson ImmunoResearch). DNA was stained with DAPI at 1:1000 (stock concentration 1 mg/ml), with the addition of 5 μg/ml RNAse A. Visualization of fixed cells was performed using a Delta Vision Core System (Applied Precision) using a 100x UplanSApo objective and a cascade 1 K EMCCD camera (Photometrics). Images were acquired as a series of z-sections separated by 0.2-μm intervals. Deconvolution was performed using the conservative ratio method in softWoRx software.

#### Drosophila embryos

*Drosophila* embryos were fixed and stained as described before^73^. Briefly, control and *san* mutant embryos were collected (0–2 or 0–6 hours after egg-laying), dechorionated with 50% bleach for 5 min, washed with water, and fixed for 40 min with gently shacking in 4 mL heptane, 0.125 mL 37% formaldehyde and 0.875 mL phosphate buffered saline (PBS, pH 7.4). After removal of aqueous phase and addition of 4 mL methanol, the embryos were vigorously shaking during 1 min for removal of embryonic vitelline membrane. Following rehydration, embryos were blocked overnight at 4 °C with PBS containing 0.1% Tween-20, 0.1% bovine serum albumin and 1% donkey serum (BBT + serum). Primary antibody incubations were done overnight in BBT + serum at 4 °C. Embryos were washed extensively in PBS containing 0.1% Tween-20 (PBT), re-blocked in BBT + serum, and incubated with the appropriate secondary antibody for 2 hours at room temperature. Used primary antibody was anti-pSer10-Histone H3 at 1:1000 (Upstate Biotechnology). Secondary antibody was anti-rabbit Alexa Fluor 488 at 1:1000 (Jackson ImmunoResearch). Embryos were extensively washed in PBT and DNA was stained with DAPI at 1:10000 (stock concentration 2 mg/ml). Embryos were mounted in Fluorescent Mounting Medium (DakoCytomation) and were visualized using a LSM710 Confocal microscope. The Z-stacks projections were obtained using Image J program (Grouped Zprojector, maximum pixel intensity).

### Biochemistry

#### Protein co-immunoprecipitation

To analyze cohesin complex composition in S2 cells, co-immunoprecipitation was done using protein extracts from S2 cells expressing or not Myc-tagged Scc1. Briefly, for S2 cells with endogenous levels of Scc1, 1 mg of protein extract was diluted in 1 ml NB buffer and incubated with rabbit anti-Scc1 (1:250 dilution) or the pre-immune (1:10,000 dilution) as control, during 1 hr at 4 °C. Subsequently, 0.9 mg of Dynabeads Protein G (Invitrogen, Grand Island, NY, USA) were added to the immune complex and incubated 1 hr at 4 °C. For S2 cells ovexpressing Myc-tagged Scc1, 1 mg of protein was diluted in 1 ml NB buffer and incubated with 0.25 mg anti-c-Myc Magnetic beads (Invitrogen, Grand Island, NY, USA) for 1 hr at 4 °C. Both Dynabeads Protein G and anti-c-Myc Magnetic beads were then washed three times with NB buffer and resuspended in 50 μl of ammonium bicarbonate (50 mM, pH 7.8). Samples were then analyzed by liquid chromatography coupled to tandem mass spectrometry (Mass Spectrometry Laboratory, Institute of Biochemistry and Biophysics, Poland).

For pull down assay from protein extracts of *san*-RNAi or *control*-RNAi treated S2 cells overexpressing GFP-tagged Smc3 (Smc3-GFP), and treated (Fig. 6B) or not (Fig. 6A) with 25 μM of colchicine for 12 hours, 1 mg of protein was diluted in 1 ml NB buffer and incubated with 0.25 mg GFP-Trap Magnetic beads (Chromotek, Grand Island, NY, USA) for 1 hr at 4 °C. Beads were then washed three times with NB buffer and than boiled in 75 μL of Laemmli buffer. The expression levels of Scc1, Dmt, San and Smc3 were than analysed by western blot analysis.

#### Mass spectrometry

Peptides mixtures were analyzed by LC-MS-MS/MS (liquid chromatography coupled to tandem mass spectrometry) using Nano-Acquity (Waters, Milford, MA, USA) LC system and Orbitrap Velos mass spectrometer (Thermo Electron Corp., San Jose, CA, USA). Prior to analysis, proteins were subjected to standard ‘in-solution digestion’ procedure, during which proteins were reduced with 100 mM DTT (for 30 min at 56 °C), alkylated with 0,5 M iodoacetamide (45 min in darkroom at room temperature), and digested overnight with trypsin (sequencing Grade Modified Trypsin-Promega V5111). The peptide mixture was applied to an RP-18 precolumn (nanoACQUITY Symmetry C18—Waters 186003514) using water containing 0,1% TFA as mobile phase, then transferred to nano-HPLC RP-18 column (nanoACQUITY BEH C18–Waters 186003545) using an acetonitrile gradient (0–35% AcN in 180 min) in the presence of 0.05% formic acid with a flow rate of 250 nl/min. The column outlet was directly coupled to the ion source of the spectrometer, operating in the regime of data dependent MS to MS/MS switch. A blank run ensuring no cross contamination from previous samples preceded each analysis.

Raw data were processed by Mascot Distiller followed by Mascot Search (Matrix Science, London, UK, on-site license) against Flybase database. Search parameters for precursor and product ions mass tolerance were 100 ppm and 0.6 Da, respectively, enzyme specificity: trypsin, missed cleavage sites allowed: 0, fixed modification of cysteine by carbamidomethylation, and variable modification of methionine oxidation. Peptides with Mascot Score exceeding the threshold value corresponding to <5% False Positive Rate, calculated by Mascot procedure, and with the Mascot score above 30 were considered to be positively identified.

Human orthologs were determined using DSRC Integrative Ortholog Prediction Tool (DIOPT) (http://www.flyrnai.org/cgi-bin/DRSC_orthologs.pl). Only scores above two were considered such as the best matches when there was more than one match per input.

#### Western blot analysis

S2 cells were collected after centrifugation (1200 rpm, 10 min at 4 °C). 0–2 hours after egg-laying *Drosophila* embryos were collected and dechorionated with 50% commercial bleach solution. Both S2 cells and embryos samples were homogenized in NB buffer (150 mM NaCl, 50 mM Tris–HCl pH 7.5, 2 mM EDTA, 0.1% NP-40, 1 mM DTT, 10 mM NaF, and EDTA-free protease inhibitor cocktail (Roche)), and centrifuged at 20000 *g* for 3 min at 4 °C. Supernatant was recovered and centrifuged twice more. Bradford protein assay (BioRad) was used to calculate extract protein concentration. Protein samples were boiled for 5 min in Laemmli buffer (Sigma) and 15 μg (per lane) was loaded onto a SDS 6% or 12% acrylamide gel. Proteins were subsequently transferred onto Hybond-ECL membranes (Amersham) and Western blotting was performed using standard procedures. Briefly, the Hybond-ECL membrane was blocked overnight in 5% non-fat milk/PBT (0.1% Tween-20, 1x PBS) at 4 °C. Primary antibodies were incubated overnight at 4 °C, with shaking. Following extensive washes with PBT, secondary antibodies were incubated for 4 hours at room temperature. After extensive washes, the proteins of interest were detected with an ECL Plus western blotting detection system (GE Healthcare). Primary antibodies used were rabbit anti-San at 1:1000 dilution, rabbit anti-Scc1 at 1:250 dilution, guinea pig anti-Smc1 at 1:500 dilution^74^, rat anti-Dmt at 1:100 dilution^75^, mouse anti-GFP (Roche) at 1:500 dilution, mouse anti-alpha-Tubulin (Dm1A) at 1:50,000 dilution (Sigma). Secondary detection was performed with anti-rabbit, anti-mouse, anti-guinea pig and anti-rat HRP-conjugated antibodies used at a final concentration of 1:5000. Uncropped images of all protein blots can be found in Supplementary Fig. 7.

#### Generated antibodies

Anti-Scc1 and Anti-San rabbit polyclonal antibodies were raised against His-tagged recombinant proteins corresponding to amino acids 80–184 of Naa50/San and 561–715 of Scc1 (Metabion international AG, Germany). Both antibodies were affinity purified. Anti-Scc1 and Anti-San antibodies were validated by western blot of protein extracts from S2 cells depleted for Scc1 (Supplementary Fig. 5) or San (Supplementary Fig. 3A), respectively.

#### Alignment of Scc1 N-terminal sequences

Protein sequence of Scc1 in *H. sapiens* was used to identify orthologs from fifteen species (7 holozoans, 2 fungi, 1 amoebozoa, 3 plants, 1 excavate and 1 chromalveolata) representative of the eukaryotic tree of life. Reciprocal bidirectional protein BLAST approach was used to retrieve the sequences from publicly available genome databases NCBI. Geneious R7 software was used with default parameters for alignment of N-terminal protein sequences.

### *In vitro* acetylation of Scc1

#### Expression and purification of MBP-San^WT^ and MBP-San^R84A Y124F^

The pETM41-*san*^*R84A Y124F*^ mutant plasmid was generated using Stratagene multisite quikchange kit with pETM41-*san*^*WT*^ plasmid as template and the primers listed in Table 2. One Shot^®^ BL21 Star^TM^ Chemically Competent *E. coli* cells were transformed with pETM41-*san*^*WT*^ or pETM41- *san*^*R84A Y124F*^ encoding the MBP-San-6xHis and the MBP-San^R84A Y124F^-6xHis proteins, and a 200 mL culture was grown to an OD_600nm_ of 0.6 (at 37 °C), followed by transfer to 18 °C and addition of IPTG to a final concentration of 1 mM. Cells were incubated at 18 °C in a shaker at 250 rpm for 18 hours, and harvested the following day by centrifugation at 3000 × *g* and 4 °C for 15 minutes. Cell pellets were resuspended in 15 mL lysis buffer (50 mM Tris-HCl, 0.3 M NaCl, 2 mM DTT, 1 tablet/50 mL Complete EDTA free protease inhibitor cocktail (Roche), pH 7.4) and applied to the French press. After French press, the cell lysate was centrifuged for 15 minutes at 15,000 × *g*. The supernatant containing the soluble protein fraction was added to a 2 × 1 mL HisTrap column (Amersham), washed with IMAC wash buffer (50 mM Tris-HCl, 0.3 M NaCl, 2 mM DTT, 20 mM imidazole, pH 7.4), and eluted with IMAC elution buffer (same as wash buffer, but with 300 mM imidazole). Fractions containing recombinant protein were combined and subjected to gel filtration on a HiLoad 16/60 Superdex 75 column (Amersham), and eluted with gel filtration buffer (50 mM Tris-HCl, 0.3 M NaCl, 1 mM DTT, pH 7.4). Protein purity was confirmed by SDS-PAGE and Coomassie staining, and concentration was checked by absorbance measurements at 280 nm.

#### DTNB based Nt-acetylation assay

DTNB reacts with free thiol groups to give the product NTB^2−^, the concentration of which can be measured spectrophotometrically. Using the method of Thompson *et al*.^76^, slightly modified^77^, we quantified the formation of NTB^2−^ after reaction of DTNB with an acetyltransferase assay sample. The time course acetyltransferase assay was performed by incubating purified MBP-San (300 nM) in acetylation buffer (50 mM Tris-HCl, 1 mM DTT, 10% glycerol, 0.2 mM EDTA, pH 8.5) with 150 μM substrate peptide (Biogenes) and 150 μM acetyl-CoA (Sigma Aldrich). Reactions were stopped with two times the volume of quenching buffer (3.2 M guanidinium-HCl, 100 mM sodium phosphate dibasic pH 6.8) after 20, 30, 40 and 60 minutes at 37 °C. For comparing San^wt^ and San^R84A Y124F^ activity, 300 nM enzyme was used in the same condition as above, only the reaction was stopped after 30 min. To measure CoA production, DTNB (2 mM final, dissolved in 100 mM sodium phosphate dibasic pH 6.8 and 10 mM EDTA) was added to the quenched reaction and the absorbance at 412 nm was measured. Thiophenolate production was quantified assuming λ = 13.7 × 103 M^−1^ cm^−1^. Background absorbances were determined and subtracted from the absorbance determined for each individual reaction. Assays were performed in triplicate and turnover for the limiting substrate did not exceed 10%.

#### Synthetic substrate peptides

Peptides were custom made (Biogenes), varying in their 7 N-terminal residues (MLGPEGG (corresponding to the N-terminus of heterogenous nuclear ribonucleoprotein F), MFYEHII (Scc1) and SESSSKS (high-mobility group protein A1), but with the same 17 C-terminal residues (RWGRPVGRRRRPVRVYP[OH]). The common C-terminal segment is identical to the adrenocorticotropic hormone, with lysines replaced by arginines to ensure that no ε-acetylation interfered with the activity measurements.

#### Statistical analysis

Unpaired *t* test and two-way ANOVA were performed using Prism V5 (GraphPad Software, San Diego, CA, USA).

## Additional Information

**How to cite this article**: Ribeiro, A. L. *et al*. Naa50/San-dependent N-terminal acetylation of Scc1 is potentially important for sister chromatid cohesion. *Sci. Rep.* **6**, 39118; doi: 10.1038/srep39118 (2016).

**Publisher's note:** Springer Nature remains neutral with regard to jurisdictional claims in published maps and institutional affiliations.

## Supplementary Material

Supplementary Movie S1

Supplementary Movie S2

Supplementary Movie S3

Supplementary Movie S4

Supplementary Movie S5

Supplementary Movie S6

Supplementary Movie S7

Supplementary Movie S8

Supplementary Movie S9

Supplementary Movie S10

Supplementary Movie S11

Supplementary Movie S12

Supplementary Movie S13

Supplementary Movie S14

Supplementary Movie S15

Supplementary Movie S16

Supplementary Information
