# Supplementary material for: Naa50/San-dependent N-terminal acetylation of Scc1 is potentially important for sister chromatid cohesion
Source: Sci Rep. 2016 Dec 20;6:39118. doi: 10.1038/srep39118 (PMC5171793; doi:10.1038/srep39118)
Supplement: Supplementary Information [file srep39118-s17.pdf]

**Naa50/San-dependent N-terminal acetylation of Scc1 is potentially important for sister chromatid cohesion.**

Ana L. Ribeiro[1,2], Rui D. Silva[1,2], Håvard Foyn[4], Margarida N. Tiago[1,2], Om S. Rathore[1,2], Thomas Arnesen[4,5], Rui Gonçalo Martinho[1,2,3,#].

**Supplementary Information:**

Supplementary Figure 1

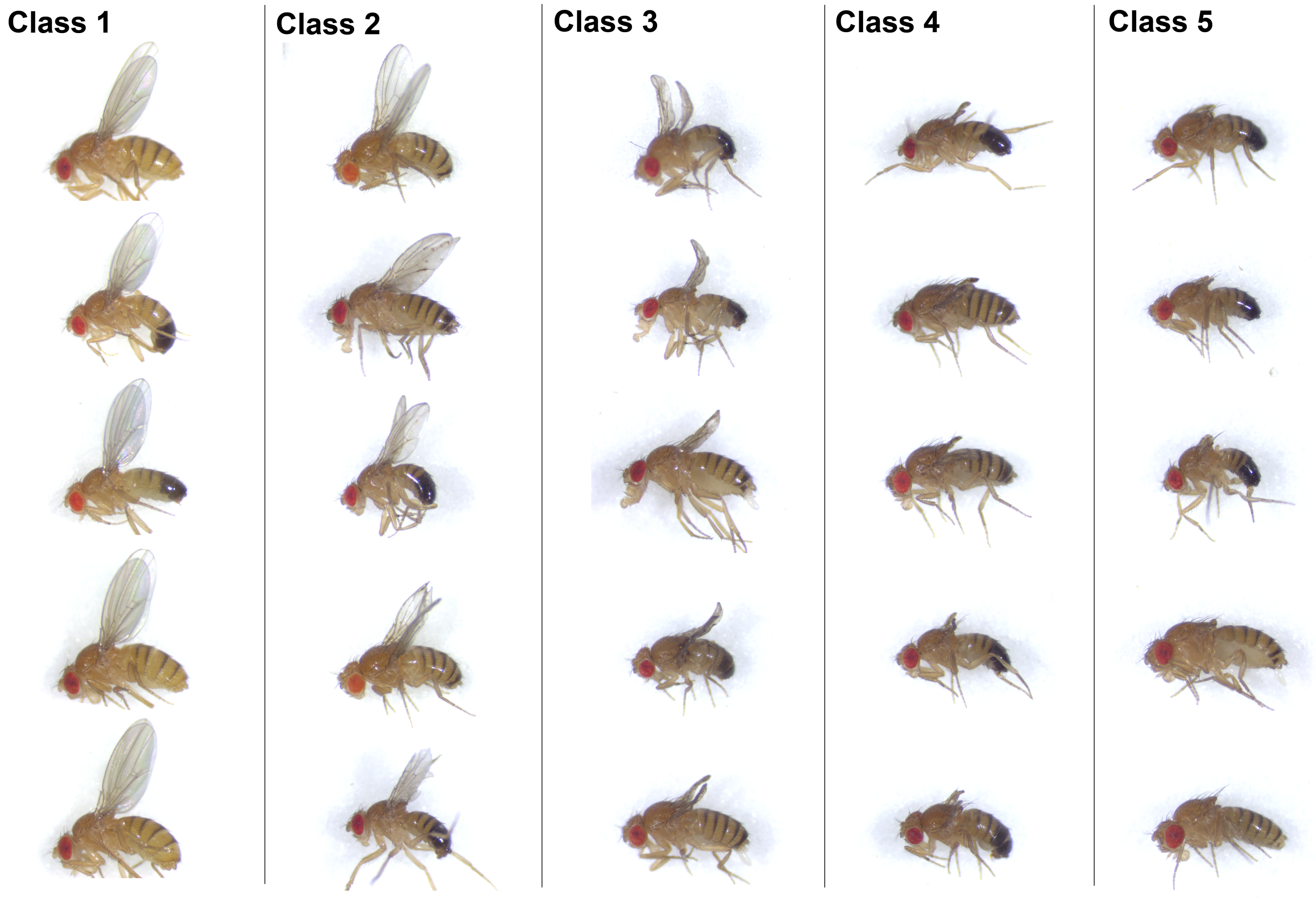

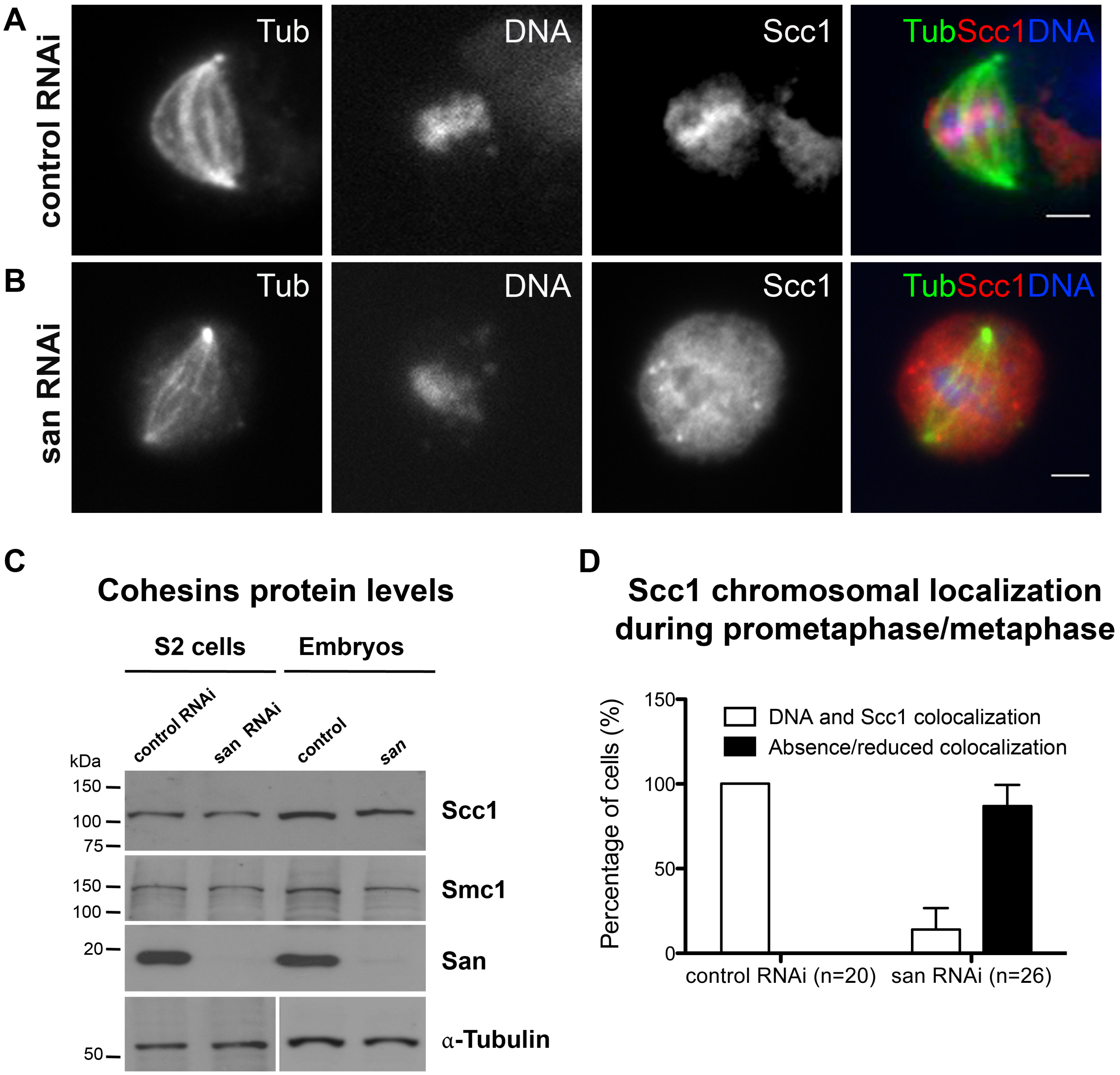
A
control RNAi
Tub
DNA
Scc1
TubScc1DNA
B
san RNAi
Tub
DNA
Scc1
TubScc1DNA
C
Cohesins protein levels
S2 cells
Embryos
control RNAi
san RNAi
control
san
kDa
150
100
75
Scc1
150
100
Smc1
20
San
50
α-Tubulin
D
Scc1 chromosomal localization
during prometaphase/metaphase
Percentage of cells (%)
150
100
50
0
DNA and Scc1 colocalization
Absence/reduced colocalization
control RNAi (n=20)
san RNAi (n=26)

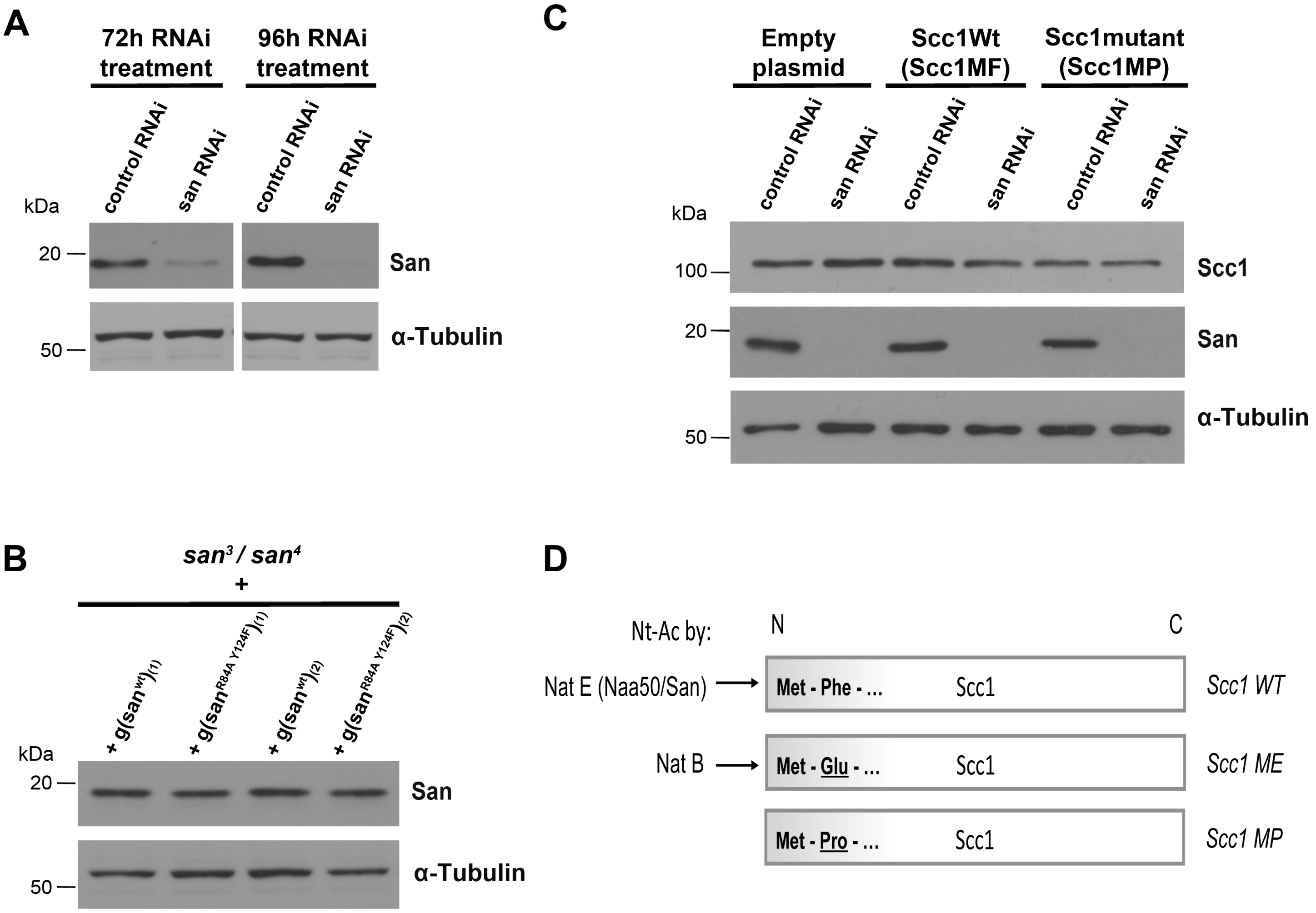

A
72h RNAi treatment
96h RNAi treatment
control RNAi
san RNAi
control RNAi
san RNAi
kDa
20
50
San
α-Tubulin
B
san3 / san4
+
+ g(sanwt)(1)
+ g(sanR84A Y124F)(1)
+ g(sanwt)(2)
+ g(sanR84A Y124F)(2)
kDa
20
50
San
α-Tubulin
C
Empty plasmid
Scc1Wt (Scc1MF)
Scc1mutant (Scc1MP)
control RNAi
san RNAi
control RNAi
san RNAi
control RNAi
san RNAi
kDa
100
20
50
Scc1
San
α-Tubulin
D
Nt-Ac by:
N
C
Nat E (Naa50/San)
Met - Phe - ...
Scc1
Scc1 WT
Nat B
Met - Glu - ...
Scc1
Scc1 ME
Met - Pro - ...
Scc1
Scc1 MP

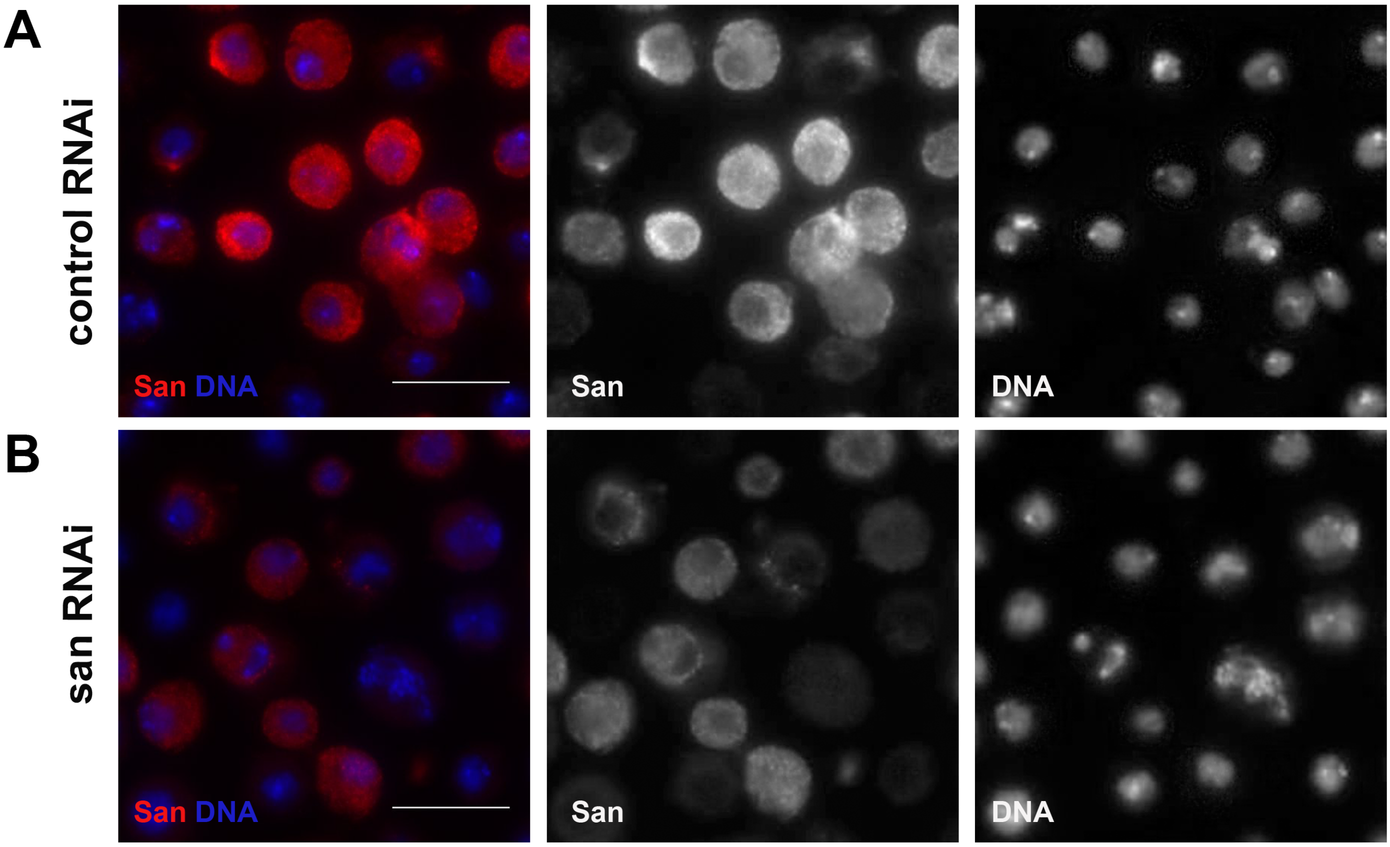
A
control RNAi
San DNA
San
DNA
B
san RNAi
San DNA
San
DNA

**A**

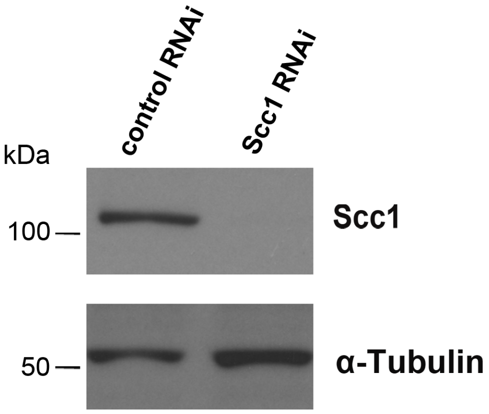

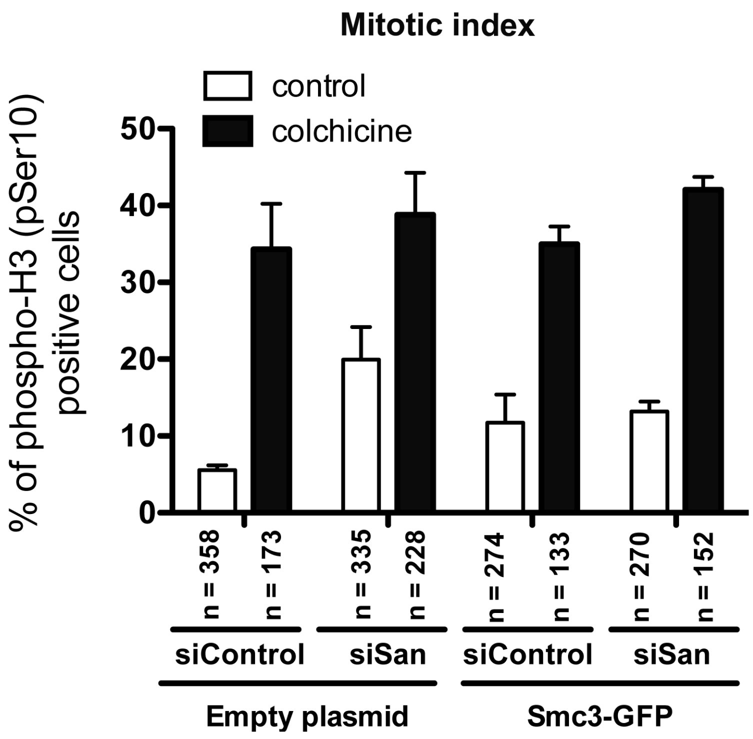
Mitotic index
control
colchicine
% of phospho-H3 (pSer10) positive cells
50
40
30
20
10
0
n = 358
n = 173
n = 335
n = 228
n = 274
n = 133
n = 270
n = 152
siControl
siSan
siControl
siSan
Empty plasmid
Smc3-GFP

**Figure 6 Dalmatian**

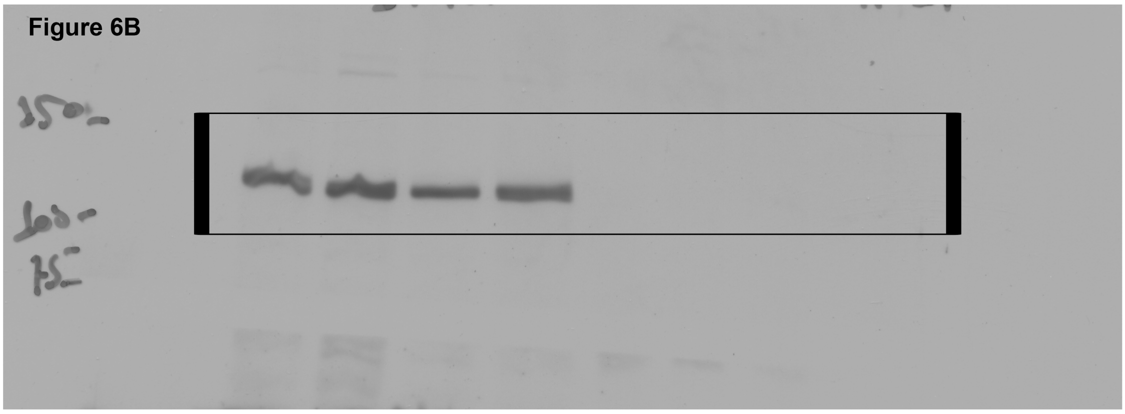


Figure 6B

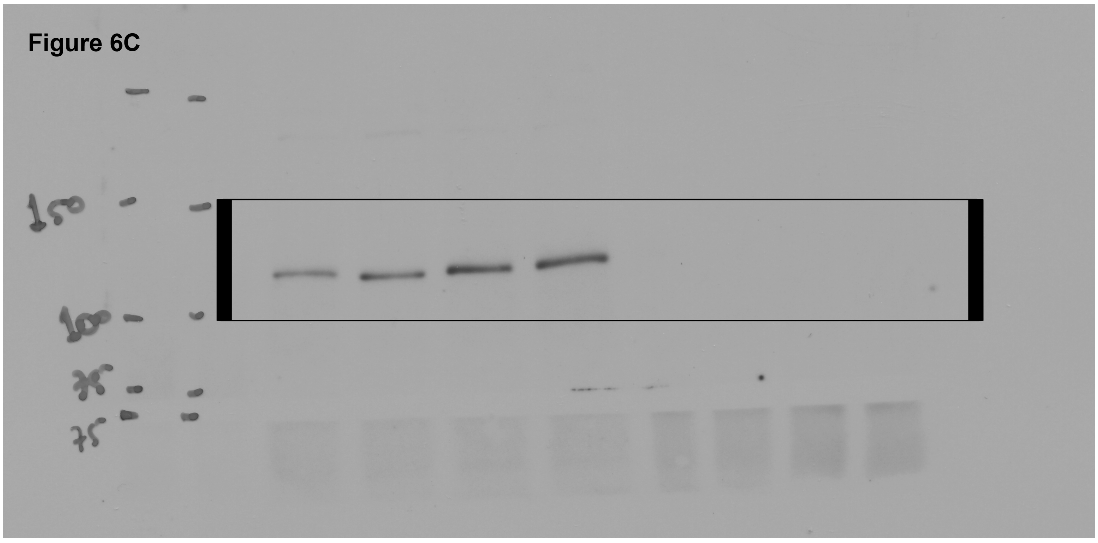


Figure 6C

Figure 6B

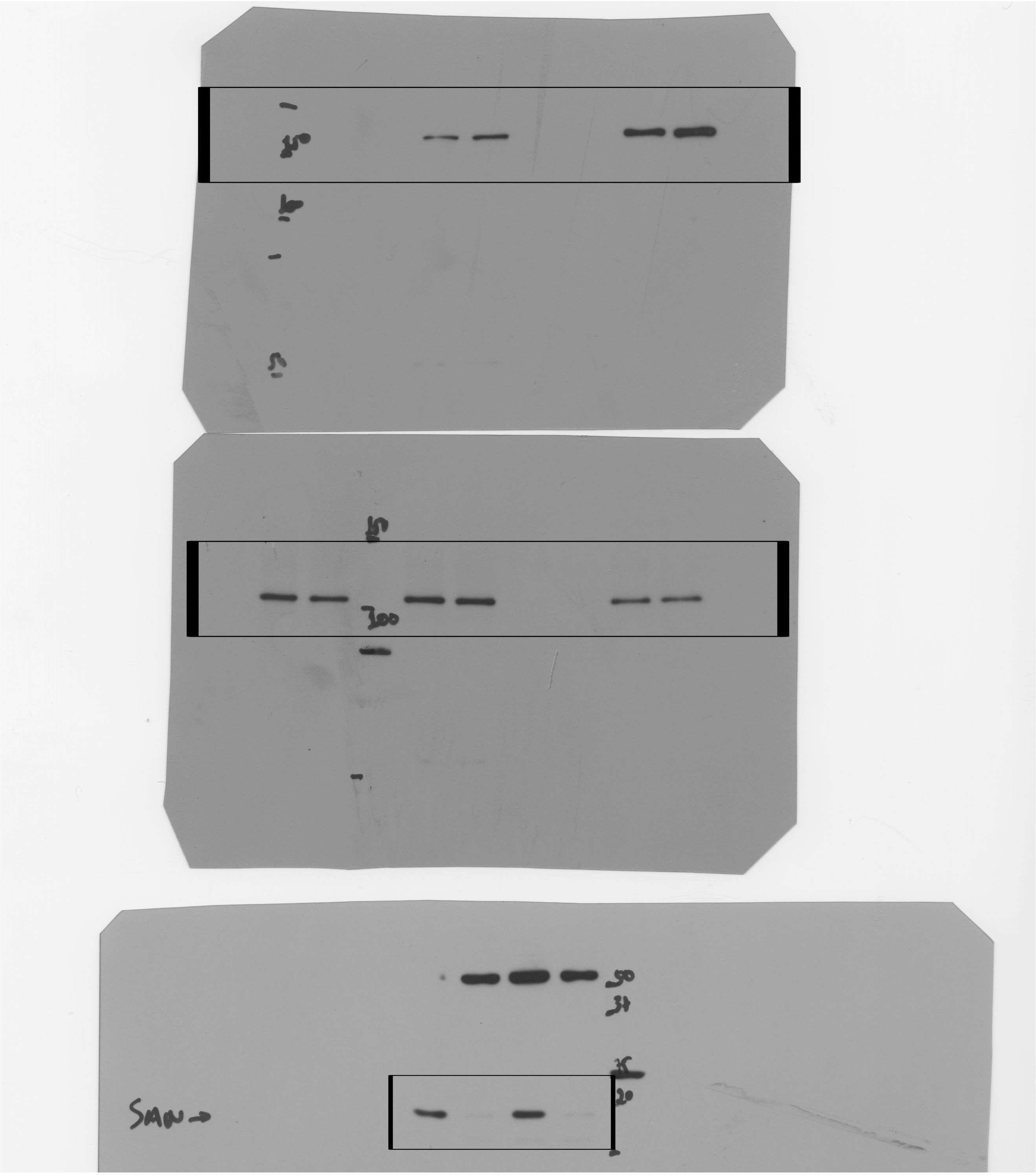

Figure 6C

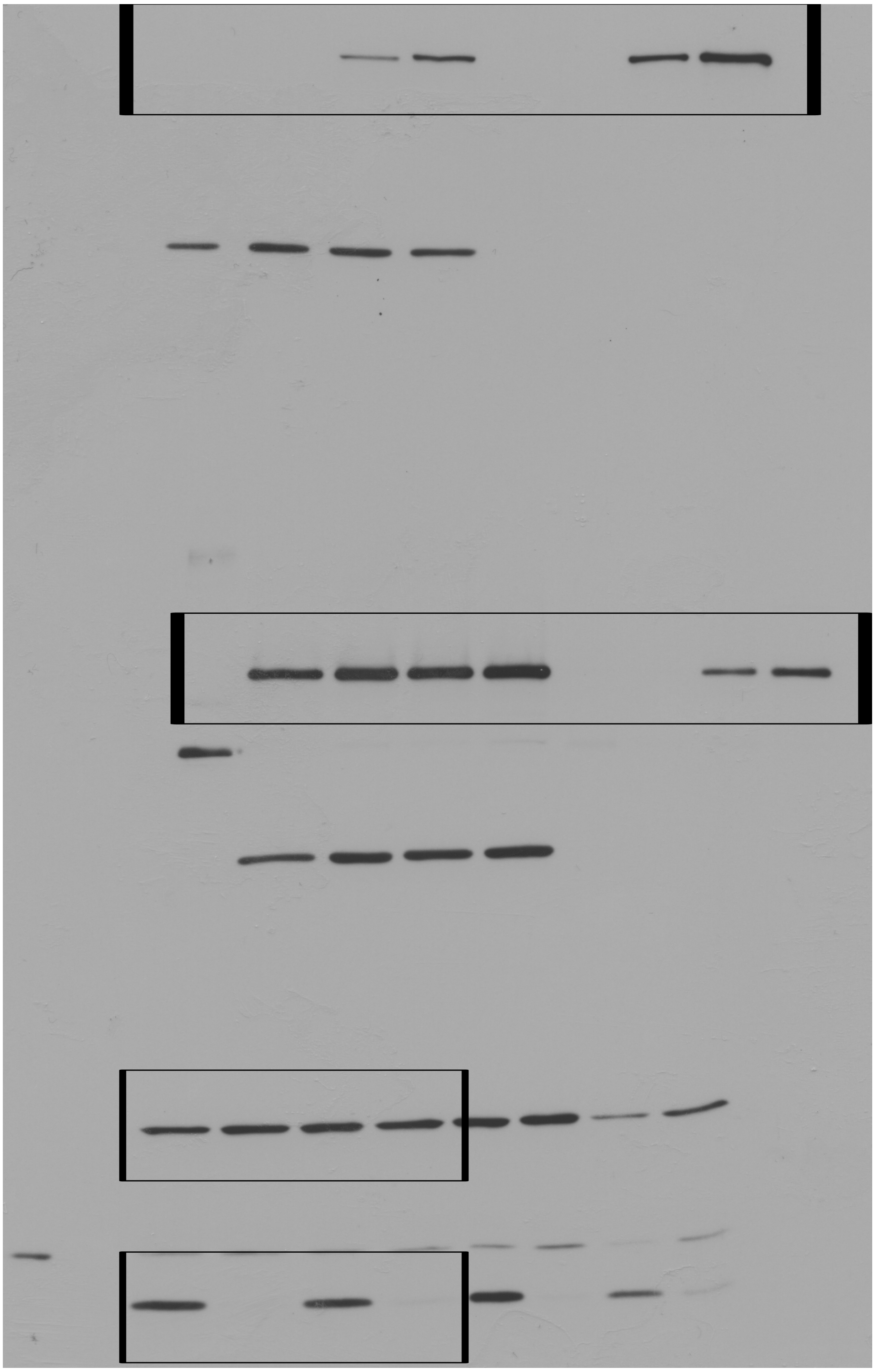

Supplementary
Fig.2C

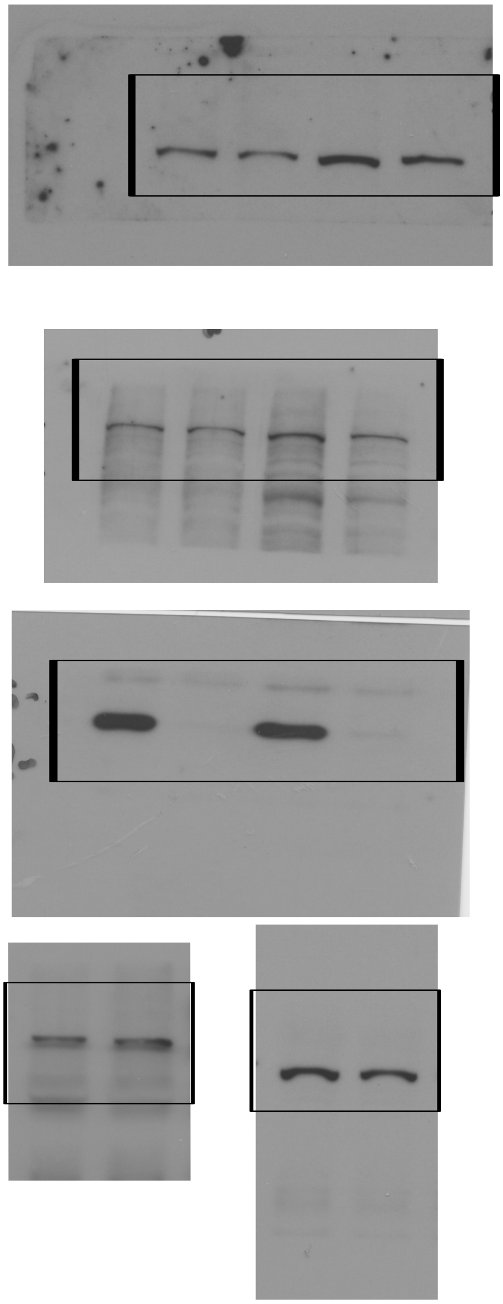

**Supplementary Fig.3A**

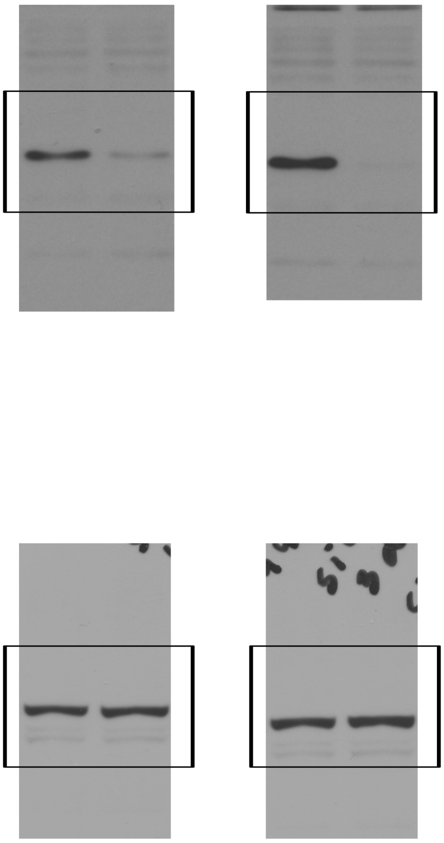

**Supplementary Fig. 3B**

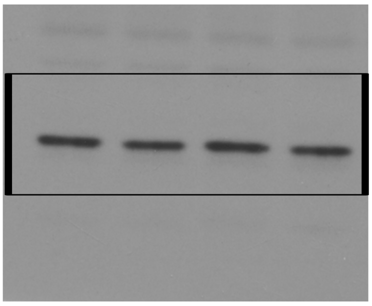

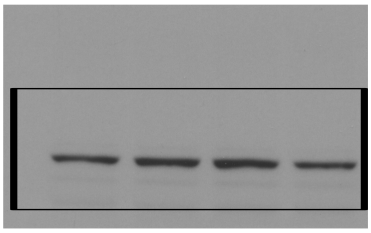

**Supplementary Fig. 3C**

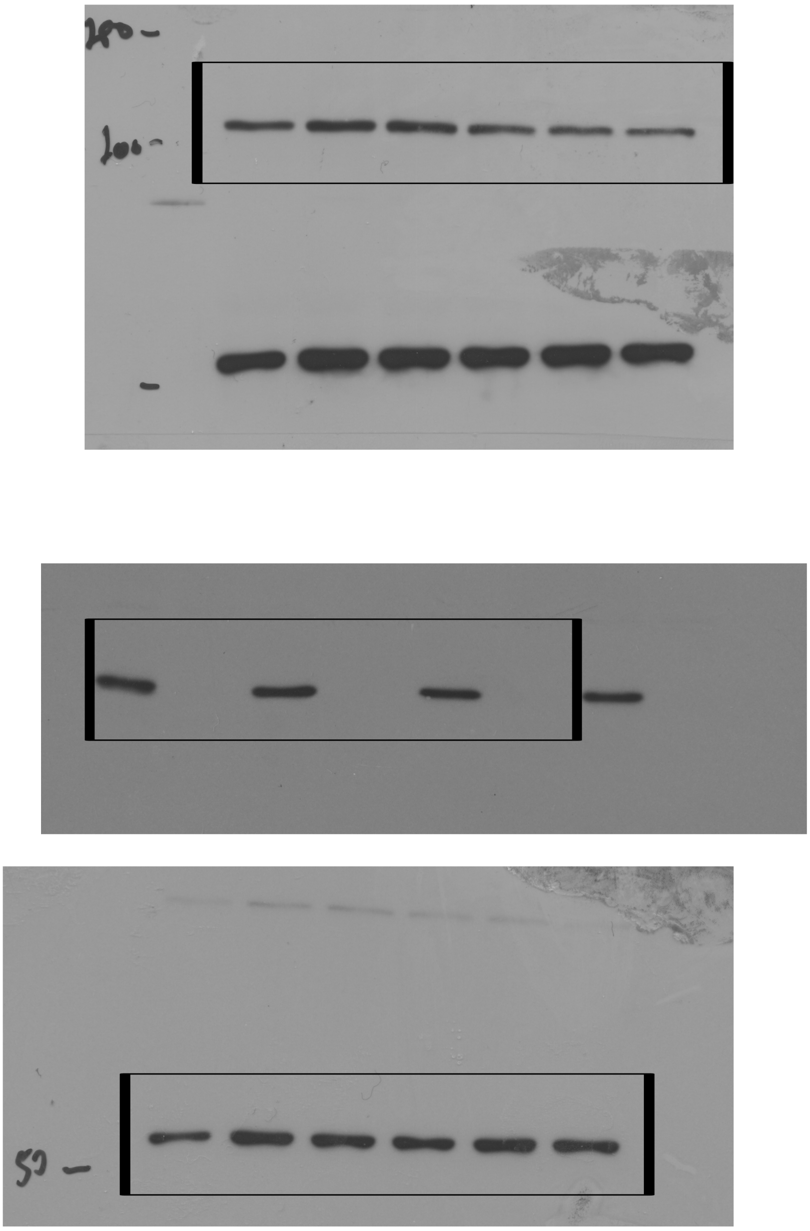

**Supplementary Fig. 5**

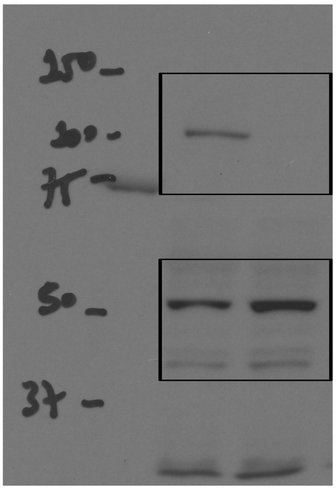

Suppl. Table 1. *Drosophila* stocks used in this study.

| Name | Genotype | Description | Source/ Reference |
|---|---|---|---|
| Oregon R (OR) | | Wild type stock. | |
| Nubbin-Gal4 | y , $w^{1118}$; P{$w^{+}$, nubbin-GAL4} | Contains the Nubbin-Gal4 driver for specific expression in the pouch of wing imaginal disc. | BDSC |
| UAS-San RNAi | y $w^{1118}$; P{KK101696, $y^{+}$, $w^{3'}$} | Contains a dsRNA under UAS control for RNAi of san. | VDRC |
| UAS-mCherry RNAi | $y^{1}$ $sc^{*}$ $v^{1}$; P{$y^{+t7.7}$ $v^{+t1.8}$=VALIUM20-mCherry}attP2 | Contains a dsRNA under UAS control for RNAi of mCherry. | BDSC/TRiP |
| UAS-deco RNAi | $y^{1}$ $sc^{*}$ $v^{1}$; P{ $y^{+t7.7}$ $v^{+t1.8}$=TRiP.GL00528}attP2 | Contains a dsRNA under UAS control for RNAi of deco. | BDSC/TRiP |
| UAS-vtd RNAi | $y^{1}$ $sc^{*}$ $v^{1}$; P{ $y^{+t7.7}$ $v^{+t1.8}$=TRiP.GL00522}attP2/TM3, $Sb^{1}$ | Contains a dsRNA under UAS control for RNAi of vtd/ scc1. | BDSC/TRiP |
| UAS-mau-2 RNAi | $y^{1}$ $sc^{*}$ $v^{1}$; P{ $y^{+t7.7}$ $v^{+t1.8}$=TRiP.HMS02374}attP2 | Contains a dsRNA under UAS control for RNAi of mau-2. | BDSC/TRiP |
| UAS-Smc3-Scc1-fusion | UAS-Smc3-Scc1-GFP fusion | Contains smc3 c-terminally fused with the N-terminus of vtd/scc1 under UAS control. | (Eichinger et al., 2013) |
| UAS-dmt | $w^{1118}$; P{$w^{+}$, UAS-Dmt} | Contains dmt/soronin under UAS control. | (Kerman and Andrew, 2010) |
| $dmt^{l184}$ | $dmt^{l184}$/Tm3 | Carries a loss of function allele of dmt/soronin. | (Kerman and Andrew, 2010) |
| Nubbin-Gal4 UAS-san RNAi | $w^{1118}$; P{$w^{+}$, nubbin-GAL4}, P{KK101696, $y^{+}$, $w^{3'}$}/ CyO | Contains the Nubbin-Gal4 driver and a dsRNA under UAS control for RNAi of san. | This study |
| *$san^{3}$* | w/w; FRT42B B16-79/ CyO | Loss of function allele of san. | Pimenta-Marques et al., 2008 |
| *$san^{4}$* | w/w; FRT42B B50-26/ CyO | Loss of function allele of san. | Pimenta-Marques et al., 2008 |
| Wild type san genomic construct in *$san^{4}$* background | w/w; FRT42B B50-26/ CyO; P{$w^{+}$=WALIUM22-San}attP2/ TM6B | Carries a loss of function allele *$san^{4}$* and a genomic construct that expresses a wild type Naa50/San under the control of its endogenous promoter. | This study |
| $san^{R84A\ Y124F}$ genomic construct in *$san^{4}$* background | w/w; FRT42B B50-26/ CyO; P{$w^{+}$=WALIUM22-S $san^{R84A\ Y124F}$ }attP2/TM6B | Carries the loss of function allele $san^{4}$ and expresses $san^{R84A\ Y124F}$ under control of the endogenous promoter. | This study |

BDSC - Bloomington Drosophila Stock Center; VDRC - Vienna Drosophila RNAi Center; TRiP - Transgenic RNAi Project.

Suppl. Table 2 – Complete list of primers used in this study.

| Primer name | Primer sequence | Comment |
|---|---|---|
| san RNAi Fw | TAATACGACTCACTATAGGGAGAA GCAGCATCGAACTGGGC | For san (CG12352) siRNA |
| san RNAi Rv | TAATACGACTCACTATAGGGAGAC GCTTATAGTATTGCTCCTTGGT | For san (CG12352) siRNA |
| sanAmp2 RNAi Fw | TAATACGACTCACTATAGGGAG ACAACACTGAGAACCAGCGGC | For san (CG12352) siRNA second amplicon |
| sanAmp2 RNAi Rv | TAATACGACTCACTATAGGGAGAC GTAGTAAAGCACGACAGTTCAC | For san (CG12352) siRNA second amplicon |
| deco RNAi Fw | GGATCCTAATACGACTCACTA TACGCTGGCCCGGATGTT | For deco (CG8598) siRNA |
| deco RNAi Rv | GGATCCTAATACGACTCACTATAC AACCTCCCGTCCACTATT | For deco (CG8598) siRNA |
| gfp RNAi Fw | TAATACGACTCACTATAGGGAGAC TTCAGCCGCTACCCC | For control siRNA |
| gfp RNAi Rv | TAATACGACTCACTATAGGGAGAT GTCGGGCAGCACG | For control siRNA |
| Scc1(Wt) Fw | GGGGACAAGTTTGTACAAAAAAGC AGGCTAAATGTTCTATGAGCACAT TATTTTGGC | For attB PCR to allow expression of native *Drosophila* Scc1 (CG17436) in pDONR221. |
| Scc1(MP-) Fw | GGGGACAAGTTTGTACAAAAAAGC AGGCTAAATGCCCTATGAGCACAT TATTTTGGC | For attB PCR to allow expression of native *Drosophila* Scc1 (CG17436) with a proline residue after the methionine in pDONR221. |
| Scc1(ME-) Fw | GGGGACAAGTTTGTACAAAAAAGC AGGCTAAATGGAGTATGAGCACAT TATTTTGGC | For attB PCR to allow expression of native *Drosophila* Scc1 (CG17436) with a glutamate residue after the methionine in pDONR221. |
| Scc1 Rv | GGGGACCACTTTGTACAAGAAAGC TGGGTCTTAAATTTTTGGGTTTTCG | For attB PCR to allow expression of *Drosophila* Scc1 (CG17436) in pDONR221. |
| a371t_Rv | CGAAACCAAACTTCTTAAAGAACT CGATGGCTCCG | To generate pETM41-san $^{R84A, Y124F}$ mutant plasmid with pETM41-san plasmid as template |
| a371t | CGGAGCCATCGAGTTCTTTAAGAA GTTTGGTTTCG | To generate pETM41-san $^{R84A, Y124F}$ mutant plasmid with pETM41-san plasmid as template |
| c250g_g251c_rev | GATGCCCAGGCGCGCGTACGGGGA GAGG | To generate pETM41-san $^{R84A, Y124F}$ mutant plasmid with pETM41-san plasmid as template |
| c250g_g251c | CCTCTCCCCGTACGCGCGCCTGGGC ATC | To generate pETM41-san $^{R84A, Y124F}$ mutant plasmid with pETM41-san plasmid as template |

Table title: **Complete list of primers**

Suppl. Table 3 – Adult *Drosophila* zygotic viability

| cross | Total number of F1 flies (n) | | *Cy+* | *Cy* |
|---|---|---|---|---|
| $\frac{w}{w}$ ; $\frac{san^4}{CyO}$ ; $\frac{\text{san genomic}^{Wt}}{\text{MKRS}}$<br>**x**<br>$\frac{w}{Y}$ ; $\frac{san^3}{CyO}$ ; $\frac{+}{+}$ | n = 173 | ***Sb*⁺** | **48 (28%)** | 125 (72%) |
| | n = 97 | ***Sb*** | 0 (0%) | 97 (100%) |
| $\frac{w}{w}$ ; $\frac{san^3}{CyO}$ ; $\frac{+}{+}$<br>**x**<br>$\frac{w}{Y}$ ; $\frac{san^4}{CyO}$ ; $\frac{\text{san genomic}^{Wt}}{\text{TM6B}}$ | n = 155 | ***Tb*⁺** | **47 (30%)** | 108 (70%) |
| | n = 77 | ***Tb*** | 0 (0%) | 77 (100%) |
| $\frac{w}{w}$ ; $\frac{san^4}{CyO}$ ; $\frac{\text{san genomic}^{R84AY124F}}{\text{TM6B}}$<br>**x**<br>$\frac{w}{Y}$ ; $\frac{san^3}{CyO}$ ; $\frac{+}{+}$ | n = 84 | ***Tb*⁺** | **0 (0%)** | 84 (100%) |
| | n = 43 | ***Tb*** | 0 (0%) | 43 (100%) |
| $\frac{w}{w}$ ; $\frac{san^3}{CyO}$ ; $\frac{+}{+}$<br>**x**<br>$\frac{w}{Y}$ ; $\frac{san^4}{CyO}$ ; $\frac{\text{san genomic}^{R84AY124F}}{\text{TM6B}}$ | n = 64 | ***Tb*⁺** | **0 (0%)** | 64 (100%) |
| | n = 77 | ***Tb*** | 0 (0%) | 77 (100%) |

# Supplementary Table 4

A

## Detailed LC-MS analysis of endogenous Scc1 co-immunoprecipitation assays.

| Proteins immunoprecipitated with endogeonus Scc1 | | | | anti-Scc1 / siGFP (control RNAi) | | | | | | anti-Scc1 / siSan (Naa50/San RNAi) | | | | | | pre-imune control (-) / siGFP (control RNAi) | | | | | | pre-imune control (-) / siSan (Naa50/San RNAi) | | | | | |
|---|---|---|---|---|---|---|---|---|---|---|---|---|---|---|---|---|---|---|---|---|---|---|---|---|---|---|---|
| | | | | Rep 1 | | | Rep 2 | | | Rep 1 | | | Rep 2 | | | Rep 1 | | | Rep 2 | | | Rep 1 | | | Rep 2 | | |
| CG | *Drosophila* | *Human* | Mass (kDa) | Score | Matches | Sequences | Score | Matches | Sequences | Score | Matches | Sequences | Score | Matches | Sequences | Score | Matches | Sequences | Score | Matches | Sequences | Score | Matches | Sequences | Score | Matches | Sequences |
| **Cohesion complex** | | | | | | | | | | | | | | | | | | | | | | | | | | | |
| CG6057-PA | SMC1 | SMC1 | 143298 | 1797 | 35 | 32 | 1280 | 29 | 28 | 1084 | 27 | 27 | 1283 | 28 | 25 | - | - | - | - | - | - | - | - | - | - | - | - |
| CG9802-PA | SMC3 | SMC3 | 140409 | 1729 | 36 | 33 | 1452 | 35 | 28 | 1702 | 35 | 28 | 1653 | 37 | 33 | - | - | - | - | - | - | - | - | - | - | - | - |
| CG3423-PA | SA | STAG1 | 130999 | 942 | 20 | 19 | 1019 | 22 | 20 | 584 | 13 | 12 | 971 | 21 | 19 | - | - | - | - | - | - | - | - | - | - | - | - |
| CG17436-PA | vtd | Rad21/Scc1 | 80033 | 711 | 15 | 14 | 534 | 12 | 12 | 676 | 14 | 14 | 531 | 11 | 11 | - | - | - | - | - | - | - | - | - | - | - | - |
| **Miscellaneous** | | | | | | | | | | | | | | | | | | | | | | | | | | | |
| CG1404-PB | Ran | RAN | 24877 | 244 | 5 | 3 | 89 | 1 | 1 | 69 | 2 | 2 | | | | - | - | - | - | - | - | - | - | - | - | - | - |
| CG31611-PA | His4 | HIST1H4 | 11374 | 210 | 4 | 4 | 123 | 2 | 2 | 182 | 4 | 4 | 128 | 3 | 3 | - | - | - | - | - | - | - | - | - | - | - | - |
| CG9888-PA | Fib | FIB | 34662 | 210 | 5 | 5 | 59 | 1 | 1 | | | | 131 | 3 | 3 | - | - | - | - | - | - | - | - | - | - | - | - |
| **Ribosomal** | | | | | | | | | | | | | | | | | | | | | | | | | | | |
| CG5502-PA | RpL4 | RPL4 | 45090 | 754 | 13 | 10 | 229 | 6 | 6 | 644 | 13 | 10 | 460 | 10 | 9 | - | - | - | - | - | - | - | - | - | - | - | - |
| CG5920-PA | RpS2 | RPS2 | 29065 | 580 | 11 | 9 | 213 | 4 | 4 | 282 | 5 | 5 | 169 | 3 | 3 | - | - | - | - | - | - | - | - | - | - | - | - |
| CG6779-PA | RpS3 | RPS3 | 27638 | 574 | 9 | 7 | 77 | 2 | 2 | 380 | 6 | 5 | 178 | 3 | 3 | - | - | - | - | - | - | - | - | - | - | - | - |
| CG1524-PB | RpS14a | RPS14 | 16301 | 566 | 9 | 7 | 115 | 1 | 1 | 287 | 4 | 4 | 216 | 4 | 4 | - | - | - | - | - | - | - | - | - | - | - | - |
| CG11276-PA | RpS4 | RPS4 | 29208 | 533 | 9 | 5 | 139 | 4 | 4 | 325 | 8 | 6 | | | | - | - | - | - | - | - | - | - | - | - | - | - |
| CG17521-PC | RpL10 | RPL10 | 25925 | 502 | 11 | 8 | 171 | 4 | 4 | 142 | 3 | 3 | 188 | 5 | 5 | - | - | - | - | - | - | - | - | - | - | - | - |
| CG7977-PA | RpL23A | RPL23A | 29445 | 490 | 9 | 7 | 156 | 3 | 3 | 246 | 6 | 6 | 132 | 3 | 2 | - | - | - | - | - | - | - | - | - | - | - | - |
| CG1883-PD | RpS7 | RPS7 | 22156 | 462 | 8 | 7 | 213 | 4 | 4 | 298 | 5 | 5 | 223 | 4 | 3 | - | - | - | - | - | - | - | - | - | - | - | - |
| CG7808-PC | RpS8 | RPS8 | 23837 | 434 | 6 | 5 | 161 | 2 | 2 | 259 | 3 | 3 | 183 | 3 | 3 | - | - | - | - | - | - | - | - | - | - | - | - |
| CG4897-PA | RpL7 | RPL7 | 29580 | 419 | 9 | 9 | 164 | 6 | 6 | 321 | 7 | 7 | 186 | 5 | 5 | - | - | - | - | - | - | - | - | - | - | - | - |
| CG3314-PA | RpL7A | RPL7A | 30851 | 418 | 9 | 9 | 168 | 3 | 3 | 378 | 8 | 7 | 243 | 5 | 5 | - | - | - | - | - | - | - | - | - | - | - | - |
| CG8415-PB | RpS23 | RPS23 | 16053 | 313 | 6 | 5 | 83 | 2 | 2 | 122 | 2 | 2 | | | | - | - | - | - | - | - | - | - | - | - | - | - |
| CG7939-PB | RpL32 | RPL32 | 16056 | 287 | 7 | 5 | 128 | 3 | 2 | 238 | 5 | 4 | 80 | 1 | 1 | - | - | - | - | - | - | - | - | - | - | - | - |
| CG17420-PA | RpL15 | RPL15 | 24402 | 285 | 6 | 5 | 90 | 2 | 2 | 354 | 8 | 7 | 198 | 5 | 5 | - | - | - | - | - | - | - | - | - | - | - | - |
| CG4651-PA | RpL13 | RPL13 | 25028 | 278 | 7 | 7 | 150 | 4 | 4 | 216 | 5 | 5 | 120 | 3 | 3 | - | - | - | - | - | - | - | - | - | - | - | - |
| CG9282-PA | RpL24 | RPL24 | 17601 | 265 | 6 | 5 | 188 | 4 | 4 | 150 | 3 | 3 | 165 | 3 | 3 | - | - | - | - | - | - | - | - | - | - | - | - |
| CG2746-PA | RpL19 | RPL19 | 24121 | 258 | 6 | 5 | 62 | 2 | 2 | 239 | 6 | 5 | 56 | 1 | 1 | - | - | - | - | - | - | - | - | - | - | - | - |
| CG4046-PA | RpS16 | RPS16 | 16856 | 257 | 6 | 6 | 80 | 2 | 2 | 181 | 4 | 4 | 83 | 2 | 2 | - | - | - | - | - | - | - | - | - | - | - | - |
| CG6510-PA | RpL28 | RPL18A | 21062 | 245 | 7 | 7 | 91 | 2 | 2 | 171 | 4 | 4 | 110 | 2 | 2 | - | - | - | - | - | - | - | - | - | - | - | - |
| CG12740-PB | RpL28 | RPL28 | 16019 | 241 | 5 | 4 | 145 | 4 | 4 | 78 | 3 | 3 | 127 | 4 | 4 | - | - | - | - | - | - | - | - | - | - | - | - |
| CG3395-PA | RpS9 | RPS9 | 22610 | 240 | 6 | 6 | 72 | 2 | 2 | 94 | 3 | 3 | 60 | 1 | 1 | - | - | - | - | - | - | - | - | - | - | - | - |
| CG8615-PA | RpL18 | RPL18 | 21789 | 211 | 5 | 5 | 185 | 6 | 5 | 180 | 3 | 3 | 82 | 2 | 2 | - | - | - | - | - | - | - | - | - | - | - | - |
| CG10944-PB | RpS6 | RPS6 | 28528 | 200 | 5 | 3 | 75 | 1 | 1 | 138 | 2 | 2 | 121 | 2 | 2 | - | - | - | - | - | - | - | - | - | - | - | - |
| CG8857-PC | RpS11 | RPS11 | 18232 | 195 | 5 | 5 | 87 | 2 | 2 | 148 | 4 | 4 | 132 | 3 | 3 | - | - | - | - | - | - | - | - | - | - | - | - |
| CG1263-PA | RpL8 | RPL8 | 27967 | 189 | 4 | 4 | 147 | 4 | 3 | 74 | 2 | 2 | 218 | 5 | 5 | - | - | - | - | - | - | - | - | - | - | - | - |
| CG6141-PA | RpL9 | RPL9 | 21516 | 173 | 3 | 3 | 71 | 1 | 1 | 128 | 3 | 3 | 41 | 1 | 1 | - | - | - | - | - | - | - | - | - | - | - | - |

B

## Detailed LC-MS analysis of Scc1-Myc co-immunoprecipitation assays.

| Proteins immunoprecipitated with Scc1-Myc | | | | anti-c-Myc Dynabeads & Scc1-Myc / siGFP (control RNAi) | | | | | | anti-c-Myc Dynabeads & Scc1-Myc / siSan (Naa50/San RNAi) | | | | | | anti-Myc & control (-) / siGFP (control RNAi) | | | | | | anti-Myc & control (-) / siSan (Naa50/San RNAi) | | | | | |
|---|---|---|---|---|---|---|---|---|---|---|---|---|---|---|---|---|---|---|---|---|---|---|---|---|---|---|---|
| | | | | Rep 1 | | | Rep 2 | | | Rep 1 | | | Rep 2 | | | Rep 1 | | | Rep 2 | | | Rep 1 | | | Rep 2 | | |
| CG | *Drosophila* | *Human* | Mass (kDa) | Score | Matches | Sequences | Score | Matches | Sequences | Score | Matches | Sequences | Score | Matches | Sequences | Score | Matches | Sequences | Score | Matches | Sequences | Score | Matches | Sequences | Score | Matches | Sequences |
| **Cohesion complex** | | | | | | | | | | | | | | | | | | | | | | | | | | | |
| CG17436-PA | vtd | RAD21 | 80033 | 592 | 13 | 11 | 1296 | 24 | 19 | 808 | 19 | 16 | 1122 | 23 | 19 | - | - | - | - | - | - | - | - | - | - | - | - |
| CG9802-PA | SMC3 | SMC3 | 140409 | 303 | 8 | 8 | 696 | 19 | 19 | 149 | 7 | 7 | 740 | 18 | 16 | - | - | - | - | - | - | - | - | - | - | - | - |
| CG3423-PA | SA | STAG1 | 130999 | 242 | 6 | 6 | 478 | 11 | 11 | 106 | 2 | 2 | 609 | 11 | 10 | - | - | - | - | - | - | - | - | - | - | - | - |
| CG6057-PA | SMC1 | SMC1 | 143298 | 160 | 5 | 5 | 830 | 20 | 20 | 118 | 4 | 4 | 793 | 20 | 18 | - | - | - | - | - | - | - | - | - | - | - | - |
| **Miscellaneous** | | | | | | | | | | | | | | | | | | | | | | | | | | | |
| CG4264-PE | Hsc70-4 | HSPA8 | 71317 | 508 | 11 | 10 | 710 | 12 | 11 | 95 | 10 | 10 | 1130 | 20 | 16 | - | - | - | - | - | - | - | - | - | - | - | - |
| CG4147-PB | Hsc70-3 | HSPA5 | 72308 | 162 | 3 | 3 | 182 | 3 | 3 | 728 | 2 | 2 | 256 | 4 | 4 | - | - | - | - | - | - | - | - | - | - | - | - |
| CG1913-PA | αTub84B | TUBA3D | 50428 | 370 | 6 | 5 | 560 | 11 | 9 | 352 | 6 | 6 | 347 | 6 | 5 | - | - | - | - | - | - | - | - | - | - | - | - |
| CG9277-PA | βTub56D | TUBB4B | 51632 | 259 | 4 | 4 | 463 | 9 | 6 | 593 | 11 | 9 | 696 | 11 | 10 | - | - | - | - | - | - | - | - | - | - | - | - |
| CG16944-PA | sesB | SLC25A4 | 33072 | 59 | 1 | 1 | 143 | 5 | 4 | 121 | 3 | 3 | 299 | 8 | 7 | - | - | - | - | - | - | - | - | - | - | - | - |
| CG1404-PB | Ran | RAN | 24877 | 79 | 1 | 1 | 40 | 1 | 1 | 50 | 1 | 1 | 39 | 1 | 1 | - | - | - | - | - | - | - | - | - | - | - | - |
| CG11154-PA | ATPsynβ | ATP5B | 54074 | 50 | 2 | 2 | 106 | 1 | 1 | | | | 135 | 2 | 2 | - | - | - | - | - | - | - | - | - | - | - | - |
| CG31611-PA | His4 | HIST1H4 | 11374 | 37 | 1 | 1 | 161 | 3 | 3 | 106 | 2 | 2 | | | | - | - | - | - | - | - | - | - | - | - | - | - |
| CG32743-PA | nonC | SMG1 | 365311 | 33 | 1 | 1 | 39 | 2 | 1 | | | | 30 | 1 | 1 | - | - | - | - | - | - | - | - | - | - | - | - |
| **Ribosomal** | | | | | | | | | | | | | | | | | | | | | | | | | | | |
| CG6779-PA | RpS3 | RPS3 | 27638 | 173 | 5 | 4 | 566 | 10 | 8 | 266 | 7 | 7 | 504 | 8 | 7 | - | - | - | - | - | - | - | - | - | - | - | - |
| CG7808-PC | RpS8 | RPS8 | 23837 | 128 | 2 | 2 | 223 | 4 | 3 | 120 | 2 | 2 | 178 | 2 | 2 | - | - | - | - | - | - | - | - | - | - | - | - |
| CG3203-PB | RpL17 | RPL17 | 21855 | 100 | 3 | 2 | 51 | 1 | 1 | 53 | 1 | 1 | 178 | 3 | 2 | - | - | - | - | - | - | - | - | - | - | - | - |
| CG1883-PD | RpS7 | RPS7 | 22156 | 98 | 2 | 2 | 219 | 6 | 6 | 104 | 2 | 2 | 125 | 1 | 1 | - | - | - | - | - | - | - | - | - | - | - | - |
| CG6684-PA | RpS25 | RPS25 | 13193 | 97 | 3 | 3 | 92 | 1 | 1 | 47 | 1 | 1 | | | | - | - | - | - | - | - | - | - | - | - | - | - |
| CG11522-PA | RpL6 | RPL6 | 27727 | 87 | 2 | 2 | 116 | 2 | 2 | 141 | 3 | 3 | 295 | 6 | 4 | - | - | - | - | - | - | - | - | - | - | - | - |
| CG9282-PA | RpL24 | RPL24 | 17601 | 87 | 1 | 1 | 44 | 2 | 2 | 90 | 1 | 1 | | | | - | - | - | - | - | - | - | - | - | - | - | - |
| CG1524-PB | RpS14a | RPS14 | 16301 | 85 | 2 | 2 | 162 | 2 | 2 | 87 | 2 | 2 | 217 | 3 | 3 | - | - | - | - | - | - | - | - | - | - | - | - |
| CG7939-PB | RpL32 | RPL32 | 16056 | 79 | 1 | 1 | 138 | 4 | 4 | 66 | 1 | 1 | 157 | 3 | 2 | - | - | - | - | - | - | - | - | - | - | - | - |
| CG17420-PA | RpL15 | RPL15 | 24402 | 74 | 2 | 2 | 55 | 1 | 1 | 108 | 2 | 2 | 58 | 1 | 1 | - | - | - | - | - | - | - | - | - | - | - | - |
| CG5920-PA | RpS2 | RPS2 | 29065 | 74 | 2 | 2 | 168 | 3 | 3 | 143 | 2 | 2 | 138 | 2 | 2 | - | - | - | - | - | - | - | - | - | - | - | - |
| CG6253-PA | RpL14 | RPL14 | 19208 | 73 | 1 | 1 | 117 | 3 | 3 | 100 | 2 | 2 | | | | - | - | - | - | - | - | - | - | - | - | - | - |
| CG17521-PC | RpL10 | RPL10 | 25925 | 70 | 1 | 1 | 120 | 3 | 3 | | | | 91 | 2 | 2 | - | - | - | - | - | - | - | - | - | - | - | - |
| CG6846-PA | RpL26 | RPL26 | 17270 | 68 | 1 | 1 | 114 | 4 | 3 | | | | | | | - | - | - | - | - | - | - | - | - | - | - | - |
| CG10423-PA | RpS27 | RPS27 | 9634 | 66 | 2 | 1 | 119 | 3 | 2 | | | | 65 | 2 | 2 | - | - | - | - | - | - | - | - | - | - | - | - |
| CG11276-PA | RpS4 | RPS4X | 29208 | 60 | 1 | 1 | 76 | 3 | 3 | 84 | 2 | 2 | 54 | 2 | 2 | - | - | - | - | - | - | - | - | - | - | - | - |
| CG8857-PC | RpS11 | RPS11 | 18232 | 56 | 2 | 2 | 174 | 5 | 5 | 45 | 1 | 1 | 98 | 3 | 3 | - | - | - | - | - | - | - | - | - | - | - | - |
| CG3922-PB | RpS17 | RPS17 | 15321 | 55 | 1 | 1 | 125 | 4 | 3 | | | | 76 | 1 | 1 | - | - | - | - | - | - | - | - | - | - | - | - |
| CG7434-PA | RpL22 | RPL22 | 30638 | 55 | 1 | 1 | 167 | 2 | 2 | 81 | 2 | 2 | | | | - | - | - | - | - | - | - | - | - | - | - | - |
| CG10305-PB | RpS26 | RPS26 | 13488 | 54 | 1 | 1 | 74 | 1 | 1 | 358 | 1 | 1 | 65 | 1 | 1 | - | - | - | - | - | - | - | - | - | - | - | - |
| CG4651-PA | RpL13 | RPL13 | 25028 | 53 | 1 | 1 | 138 | 3 | 3 | 107 | 3 | 3 | 120 | 3 | 3 | - | - | - | - | - | - | - | - | - | - | - | - |
| CG15442-PC | RpL27A | MRPL27 | 17057 | 52 | 4 | 3 | 115 | 5 | 4 | 34 | 1 | 1 | 64 | 2 | 2 | - | - | - | - | - | - | - | - | - | - | - | - |
| CG7977-PA | RpL23A | RPL23A | 29445 | 47 | 1 | 1 | 162 | 3 | 3 | 151 | 3 | 3 | 104 | 2 | 2 | - | - | - | - | - | - | - | - | - | - | - | - |
| CG5502-PA | RpL4 | RPL4 | 45090 | 47 | 1 | 1 | 193 | 4 | 4 | 124 | 4 | 4 | 78 | 2 | 2 | - | - | - | - | - | - | - | - | - | - | - | - |
| CG4863-PA | RpL3 | RPL3 | 47208 | 37 | 1 | 1 | 47 | 1 | 1 | 45 | 1 | 1 | 143 | 3 | 3 | - | - | - | - | - | - | - | - | - | - | - | - |
| CG8415-PB | RpS23 | RPS23 | 16053 | 34 | 1 | 1 | 172 | 4 | 4 | | | | | | | - | - | - | - | - | - | - | - | - | - | - | - |

**Supplementary Figure 1. Examples of the scored adult wing phenotypic classes.** Class 1 only includes flies with wild type wings, class 2 includes flies with weak wing phenotype, class 3 includes flies with *san* RNAi-like wing phenotype, class 4 includes highly abnormal wings and class 5 only includes flies with absent or vestigial adult wings.

**Supplementary Figure 2. Naa50/San is required for chromosomal localization of Scc1 in *Drosophila* S2 cells.**
Scc1 association to chromosomes, but not its protein stability, is impaired after depletion of Naa50/San **(A-D).** Representative images of *Drosophila* S2 cells analyzed 96 hours after control RNAi **(A)** or *san* RNAi **(B)**. S2 cells were stained for DNA (blue), Scc1 (red) and $\alpha$-Tubulin (green). Scc1 colocalized with DNA during prometaphase/metaphase in control RNAi-treated cells **(A),** but not in *san* RNAi-treated cells **(B)**. **(C)** Western blot of total protein extracts from *san* RNAi-treated S2 cells and embryos mutant for *san* (maternal mutants) showed no clear reduction of Scc1 and Smc1 protein levels. α-Tubulin was used as a loading control. **(D)** Quantification of Scc1 mislocalization phenotype during prometaphase/metaphase after depletion of Naa50/San. Reduced colocalization between Scc1 and DNA: control RNAi-treated S2 cells (0%, n=20), *san* RNAi-treated S2 cells 86.9 % ± 10.2 (n=26). Scale bars equal 5 µm.

**Supplementary Figure 3. Naa50/San is efficiently depleted by *san* RNAi-treatment in *Drosophila* S2 cells. Absence of Naa50/San in embryos mutant for *san* (maternal mutants). Naa50/San expression levels are identical for a wild type ($San^{wt}$) and a catalyticaly dead-version ($San^{R84A\ Y124F}$). Levels of Scc1 after ectopic expression of Scc1MF (wild-type) or Scc1MP are equivalent.**
**(A)** Western blot analysis of total protein extracts from *Drosophila* S2 cells treated with *san* RNAi for 72 hours (left) and 96 hours (right), showed reduced levels of Naa50/San protein when compared with control RNAi-treated cells. Depletion of Naa50/San was more efficient 96 hours after *san* RNAi-treatment.

α-Tubulin was used as loading control. **(B)** Wild type and catalytically dead genomic constructs expressed similar levels of Naa50/San. Western blot analysis of total protein extracts from *san* mutant embryos with one copy of a wild type genomic construct ($san^{wt}$), and *san* mutant embryos with one copy of a catalytically dead genomic construct ($san^{R84A\ Y124F}$). α-Tubulin was used as loading control. *san* mutant embryos (maternal mutants) were obtained after the induction of germ line clones using the FRT/$ovo^{D}$ system{Chou, 1992 #67} and a previously published loss-of-function allele of *san* ($san^{3}$){Pimenta-Marques, 2008 #5}. Both wild type ($san^{wt}$) and catalytically dead genomic constructs ($san^{R84A\ Y124F}$) contain the gene endogenous promoter and were integrated in the same attP2 site (for more details see material and methods). [1] and [2] indicate that the genomic constructs although identical are from two independently generated *Drosophila* stocks. **(C)** Western blot analysis of total protein extracts from control RNAi and *san* RNAi-treated S2 cells, transfected with an empty plasmid (pHW), wild type Scc1 (Scc1Wt), or a non acetylatable mutant Scc1 (Scc1MP), showed identical levels of Scc1. There were no reproducible variations of Scc1 protein levels for any of the tested mutant alleles. A reduction in Naa50/San protein levels was always observed in *san* RNAi-treated cells. α-Tubulin was used as loading control. **(D)** Representation of the different Scc1 constructs used: a NatE (Naa50/San)-like substrate (Wild-type Scc1; Scc1Wt), a NatB-like substrate Scc1 (Scc1ME mutant variant), and a non-acetylatable mutant Scc1 (Scc1MP mutant variant).

**Supplementary Figure 4. Naa50/San is not enriched in the nucleus of *Drosophila* S2 cells. (A and B).** Representative images of *Drosophila* S2 cells analyzed 96 hours after control RNAi **(A)** or *san* RNAi **(B)**. S2 cells were stained for DNA (blue) and San (red). San is not enriched in the nucleus during interphase **(A)**{Williams, 2003 #4;Aksnes, 2015 #200}**.** In *san* RNAi-treated cells there is, as expected, a reduction of San protein levels which confirms signal specificity **(B)**. Scale bars equal 5 µm.

**Supplementary Figure 5. Scc1 antibody is specific for Scc1 protein. (A)** Western blot analysis of total protein extracts from *Drosophila* S2 cells treated with control RNAi (left) or *scc1* RNAi for 96 hours (right). *scc1*-RNAi treated cells showed reduced levels of Scc1 protein when compared with control RNAi-treated cells. α-Tubulin was used as loading control.

**Supplementary Figure 6. The mitotic index of cells treated with colchicine is significantly increased.** Mitotic index (% of phospho-H3 (pSer10) positive cells) for control RNAi and *san* RNAi-treated cells (96 hours after RNAi-treatment) transfected with an empty plasmid or a GFP-tagged Smc3 was, respectively, 5.6% ± 0.6 (n=358), 19.9% ± 4.2 (n=335), 11.7% ± 3.6 (n=274) and 13.2% ± 1.3 (n=270). The mitotic index for control RNAi and *san* RNAi-treated cells transfected with an empty plasmid or a GFP-tagged Smc3 and treated with 25μM of colchicine for 12 hours was, respectively, 34.3% ± 5.9 (n=173), 38.8% ± 5.5 (n=228), 34.9% ± 2.2 (n=133) and 42.1% ± 1.7 (n=152).

**Supplementary Figure 7. Uncropped images of all protein blots shown in this manuscript.**

**Supplementary Table 1. *Drosophila* stocks used in this study.**

**Supplementary Table 2. Complete list of primers used in this study.**

**Supplementary Table 3. Naa50/San catalytic activity is essential for adult *Drosophila* viability.** A genomic construct carrying a wild type copy of *san* (*san*$^{Wt}$), but not a catalytically dead allele of *san* (*san*$^{R84A\ Y124F}$), efficiently rescued the zygotic lethality of two loss-of-function alleles of *san* (*san*$^{3}$ and *san*$^{4}$){Pimenta-Marques, 2008 #5}. Given the indicated crosses, and since *Drosophila* flies homozygous for the CyO balancers are not viable, full complementation of *san* mutant viability corresponds to 33% of the total number of progeny (F1) *Drosophila* flies with the genomic transgene.

**Supplementary Table 4. Detailed LC-MS analysis of endogenous Scc1 and Scc1-Myc tagged co-immunoprecipitation assays.**

All subunits of the cohesin complex, but not Dalmatian/Soronin, were efficiently immunoprecipitated with endogenous Scc1 **(A)** or with Myc-tagged Scc1 **(B)** after depletion of Naa50/San. Co-immunoprecipitations with an anti-Scc1 antibody or with anti-c-Myc Magnetic beads (Invitrogen, Grand Island, NY, USA) were performed, respectively, using total protein extracts from *Drosophila* S2 cells or expressing a Myc-tagged Scc1. Both sets of cells were either treated with control RNAi or *san* RNAi before immunoprecipitation. Score, number of detected peptides (matches) and non-repeated peptides (sequences) for each immunoprecipitated protein is indicated. None of the proteins shown in this table were detected in the negative controls (respectively, **(A)** pre-immune serum or **(B)** *Drosophila* S2 cells expressing an empty plasmid). Two biological replicas (Rep1 and Rep2) are shown for each experimental condition.

**Movie S1. Control S2 cells (96h RNAi treatment), GFP-Histone H2B (green) and α-Tubulin-mCherry (red).**

Time-Lapse microscopy of S2 cells stably expressing GFP-Histone H2B (green) and α-Tubulin-mCherry (red) after 96 hours of control RNAi treatment. For more experimental details please see methods.

**Movie S2. San depleted S2 cells (96h RNAi treatment), GFP-Histone H2B (green) and α-Tubulin-mCherry (red).**

Time-Lapse microscopy of S2 cells stably expressing GFP-Histone H2B (green) and α-Tubulin-mCherry (red) after 96 hours of *san* RNAi treatment. For more experimental details please see methods. The arrowhead is labeling a single chromatid at the different time points.

**Movie S3. San depleted S2 cells (96h RNAi treatment), GFP-Histone H2B (green) and α-Tubulin-mCherry (red).**

Time-Lapse microscopy of S2 cells stably expressing GFP-Histone H2B (green) and $\alpha$-Tubulin-mCherry (red) after 96 hours of *san* RNAi treatment. For more experimental details please see methods. The arrowhead is labeling a single chromatid at the different time points.

**Movie S4. San depleted S2 cells (96h RNAi treatment), GFP-Histone H2B (green) and $\alpha$-Tubulin-mCherry (red), using a distinct non-overlapping dsRNA.**

Time-Lapse microscopy of S2 cells stably expressing GFP-Histone H2B (green) and $\alpha$-Tubulin-mCherry (red) after 96 hours of *san* RNAi treatment with a non-overlapping dsRNA. For more experimental details please see methods. The arrowhead is labeling a single chromatid at the different time points.

**Movie S5. Control S2 cells (96h RNAi treatment), GFP-$\alpha$-Tubulin (green) and CID-mCherry (red).** Time-Lapse microscopy of S2 cells stably expressing GFP-$\alpha$-Tubulin (green) and CID-mCherry (red) after 96 hours of control RNAi treatment. For more experimental details please see methods.

**Movie S6. San depleted S2 cells (96h RNAi treatment), GFP-$\alpha$-Tubulin (green) and CID-mCherry (red).** Time-Lapse microscopy of S2 cells stably expressing GFP-$\alpha$-Tubulin (green) and CID-mCherry (red) after 96 hours of *san* RNAi treatment. For more experimental details please see methods. The arrowhead is labeling a single chromatid at the different time points.

**Movie S7. Control S2 cells (72h RNAi treatment), GFP-Histone H2B (green) and $\alpha$-Tubulin-mCherry (red).**

Time-Lapse microscopy of S2 cells stably expressing GFP-Histone H2B (green) and $\alpha$-Tubulin-mCherry (red) after 72 hours of control RNAi treatment. For more experimental details please see methods.

**Movie S8. Deco depleted S2 cells (72h RNAi treatment), GFP-Histone H2B (green) and α-Tubulin-mCherry (red).** Time-Lapse microscopy of S2 cells stably expressing GFP-Histone H2B (green) and α-Tubulin-mCherry (red) after 72 hours of *deco* RNAi treatment. For more experimental details please see methods.

**Movie S9. San depleted S2 cells (72h RNAi treatment), GFP-Histone H2B (green) and α-Tubulin-mCherry (red).** Time-Lapse microscopy of S2 cells stably expressing GFP-Histone H2B (green) and α-Tubulin-mCherry (red) after 72 hours of *san* RNAi treatment. For more experimental details please see methods.

**Movie S10. San and Deco co-depleted S2 cells (72h RNAi treatment), GFP-Histone H2B (green) and α-Tubulin-mCherry (red).** Time-Lapse microscopy of S2 cells stably expressing GFP-Histone H2B (green) and α-Tubulin-mCherry (red) after 72 hours of *san* and *deco* RNAi co-treatment. For more experimental details please see methods. The arrowhead is labeling a single chromatid at the different time points.

**Movie S11. Control S2 cells, GFP-Histone H2B (green) and α-Tubulin-mCherry (red), transfected with pHW.**

Time-Lapse microscopy of S2 cells stably expressing GFP-Histone H2B (green) and α-Tubulin-mCherry (red), transfected with pHW (empty plasmid), and treated with control RNAi for 96 hours. For more experimental details please see methods.

**Movie S12. San depleted S2 cells, GFP-Histone H2B (green) and α-Tubulin-mCherry (red), transfected with pHW.**

Time-Lapse microscopy of S2 cells stably expressing GFP-Histone H2B (green) and α-Tubulin-mCherry (red), transfected with pHW (empty plasmid), and treated with *san* RNAi for 96 hours. For more experimental details please

see methods. The arrowhead is labeling a single chromatid at the different time points.

**Movie S13. Control S2 cells, GFP-Histone H2B (green) and α-Tubulin-mCherry (red), transfected with pHW(Scc1Wt).** Time-Lapse microscopy of S2 cells stably expressing GFP-Histone H2B (green) and α-Tubulin-mCherry (red), transfected with Scc1Wt, and treated with control RNAi for 96 hours. For more experimental details please see methods.

**Movie S14. San depleted S2 cells, GFP-Histone H2B (green) and α-Tubulin-mCherry (red), transfected with pHW(Scc1Wt).** Time-Lapse microscopy of S2 cells stably expressing GFP-Histone H2B (green) and α-Tubulin-mCherry (red), transfected with Scc1Wt, and treated with *san* RNAi for 96 hours. For more experimental details please see methods.

**Movie S15. Control S2 cells, GFP-Histone H2B (green) and α-Tubulin-mCherry (red), transfected with pHW(Scc1MP).** Time-Lapse microscopy of S2 cells stably expressing GFP-Histone H2B (green) and α-Tubulin-mCherry (red), transfected with Scc1MP, and treated with control RNAi for 96 hours. For more experimental details please see methods. The arrowhead is labeling a single chromatid at the different time points.

**Movie S16. San depleted S2 cells, GFP-Histone H2B (green) and α-Tubulin-mCherry (red), transfected with pHW(Scc1MP).** Time-Lapse microscopy of S2 cells stably expressing GFP-Histone H2B (green) and α-Tubulin-mCherry (red), transfected with Scc1MP, and treated with *san* RNAi for 96 hours. For more experimental details please see methods. The arrowhead is labeling a single chromatid at the different time points.
